# Correction: An AP Endonuclease Functions in Active DNA Demethylation and Gene Imprinting in *Arabidopsis*

**DOI:** 10.1371/journal.pgen.1005198

**Published:** 2015-04-27

**Authors:** The PLOS Genetics Staff

## Notice of Republication

This article was republished on April 7th, 2015, to correct an error in the wording of the title that was introduced during the typesetting process. The title read “An AP Endonuclease Functions in Active DNA Dimethylation and Gene Imprinting in *Arabidopsis*” whereas it should have read “An AP Endonuclease Functions in Active DNA Demethylation and Gene Imprinting in *Arabidopsis*”. The publisher apologizes for this error. Please download this article again to view the correct version. The originally published, uncorrected article and the republished, corrected article are provided here for reference.

## Supporting Information

S1 File Originally published, uncorrected article. (PDF)

S2 File Republished corrected article. (PDF)
