# Supplementary figures and images for: An AP Endonuclease Functions in Active DNA Demethylation and Gene Imprinting in Arabidopsis
Source: PLoS Genet. 2015 Jan 8;11(1):e1004905. doi: 10.1371/journal.pgen.1004905 (PMC4287435; doi:10.1371/journal.pgen.1004905)

Figure S1

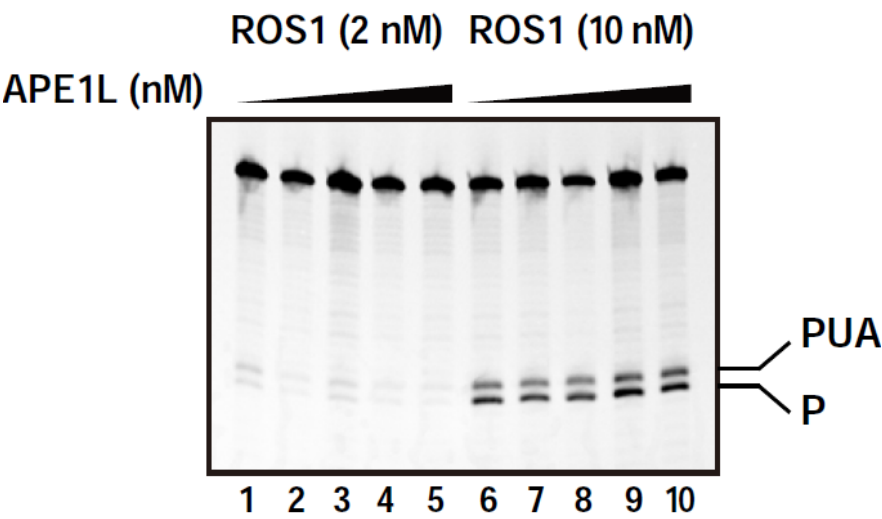

Supplement: S1 Fig — The DNA glycosylase/lyase activity of ROS1 is not increased in the presence of APE1L. Purified His-ROS1 (2 or 10 nM) was incubated in a reaction buffer lacking Mg2+ with a DNA substrate containing a 5-meC (20 nM) either in the absence or presence of purified MBP-APE1L (2, 10, 20 or 100 nM). Reactions were stopped after 16 hours and products were separated in a 12% denaturing polyacrylamide gel and detected by fluorescence scanning. (PDF) [file pgen.1004905.s001.pdf]

Figure S2

A

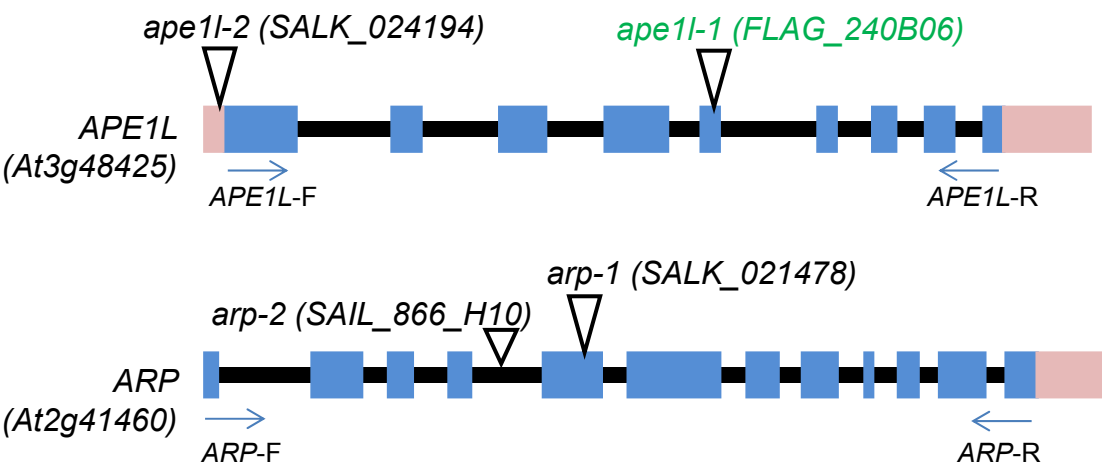

B

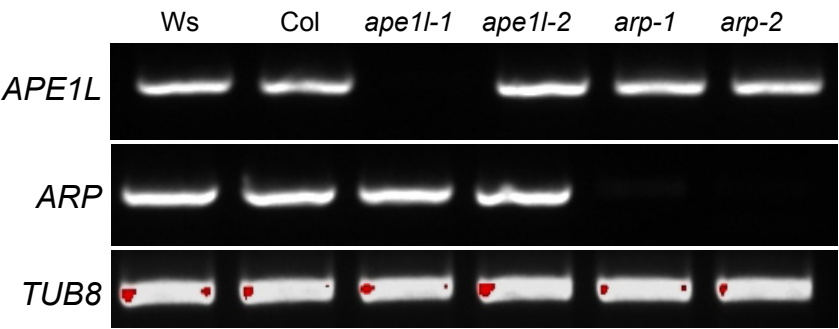

Supplement: S2 Fig — Characterization of APE T-DNA mutants. (A) Mutant lines used and the positions of T-DNA insertions in each line. The green one is from the Ws background while black ones are from the Col background. blue boxes: exon; black boxes: intron: pink boxes: UTR. (B) RT-PCR results showing undetectable expression of APE1L and ARP transcripts in ape1l-1, arp-1 and arp-2 respectively. ape1l-2 has a normal expression of APE1L. TUB8 was used as a control. (PDF) [file pgen.1004905.s002.pdf]

Figure S3

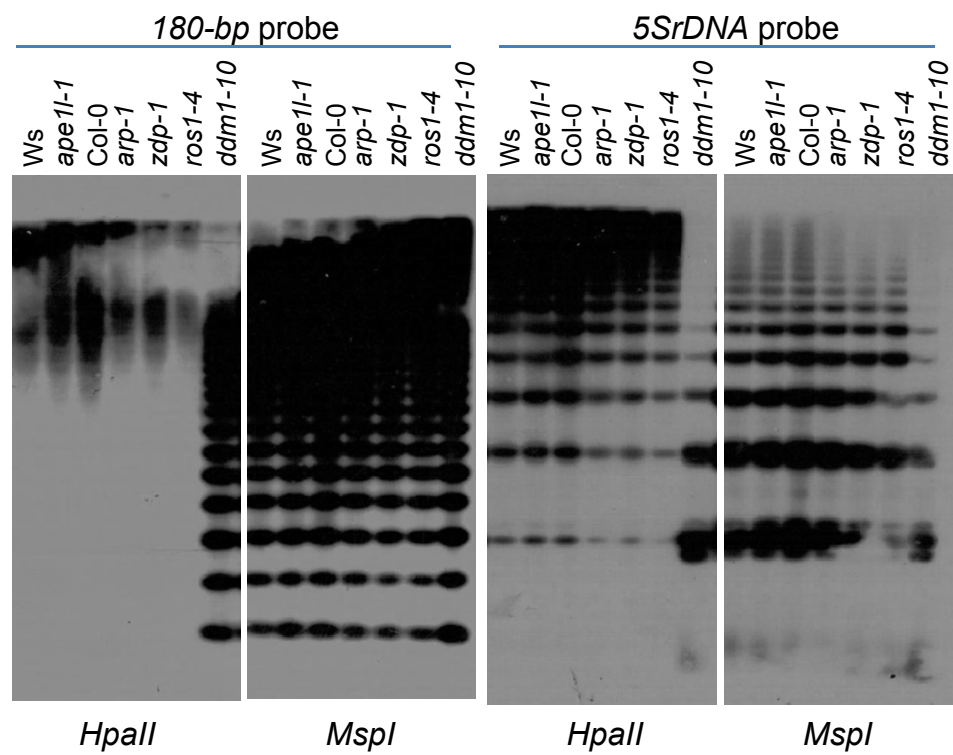

Supplement: S3 Fig — Methylation status of 5S rDNA and centromeric DNA repeats in the plants of different genotypes. Genomic DNA from plants of different genotypes was digested with the methylation sensitive enzyme HpaII (CG and CHG methylation) or MspI (CHG methylation), and hybridized with 5S rDNA, or 180-bp centromeric repeat probes. ddm1-10 was used as a hypomethylation control. (PDF) [file pgen.1004905.s003.pdf]

Figure S5

A

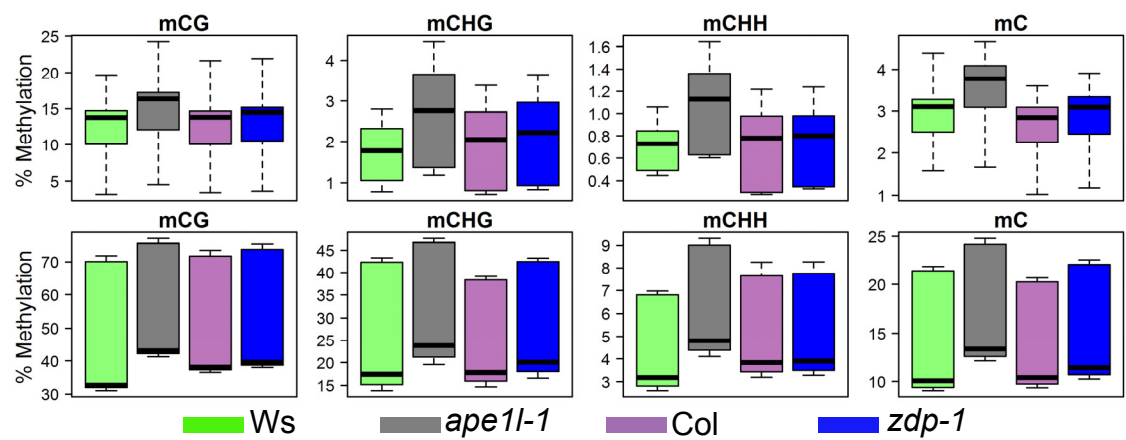

B

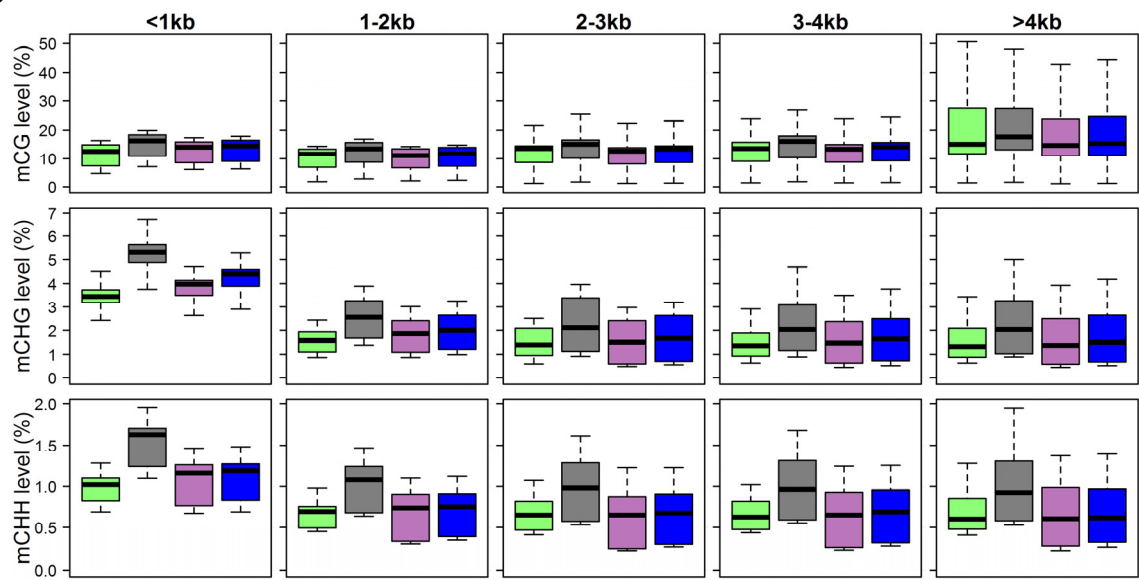

Figure S5 continued

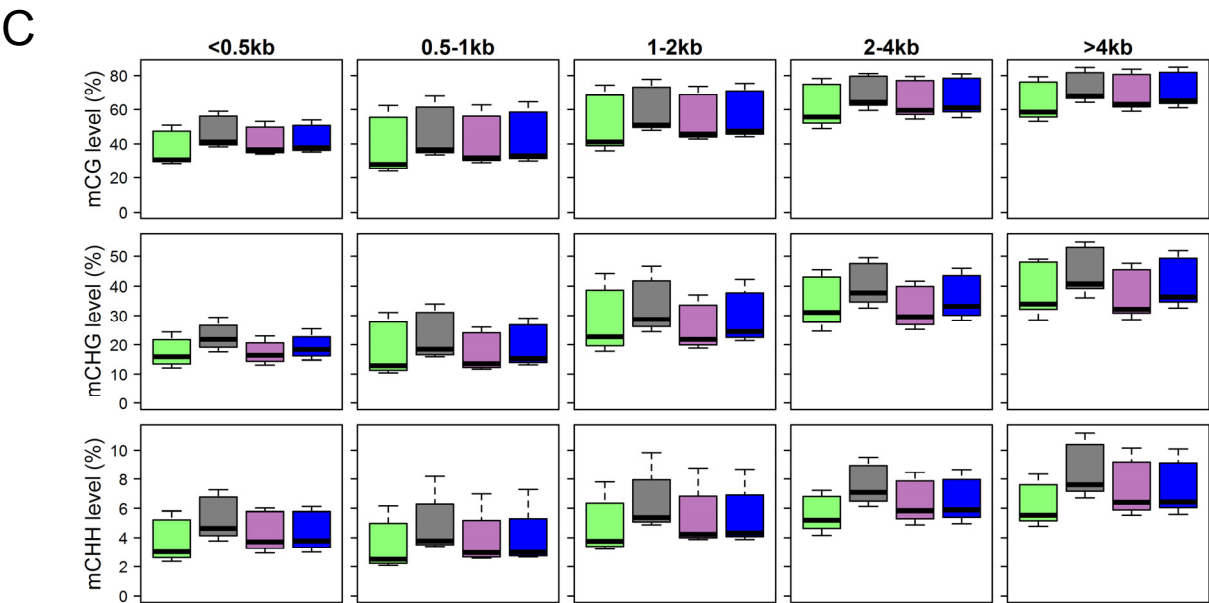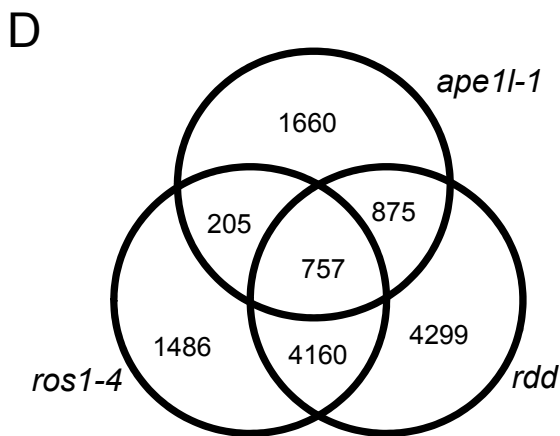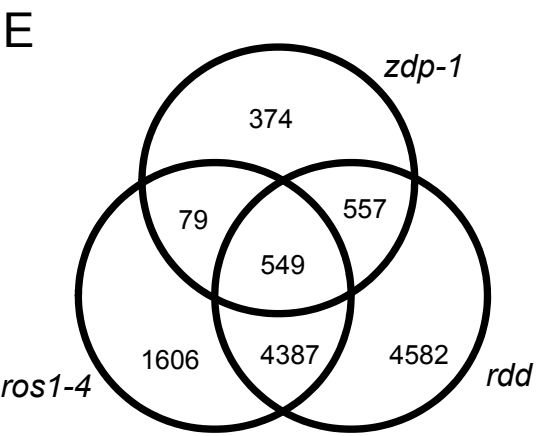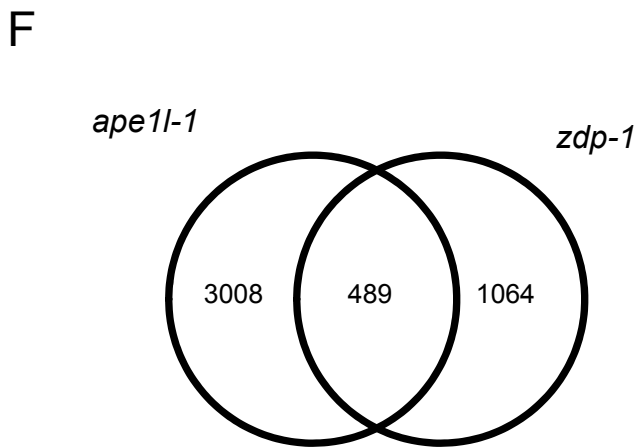

Figure S5 continued

G

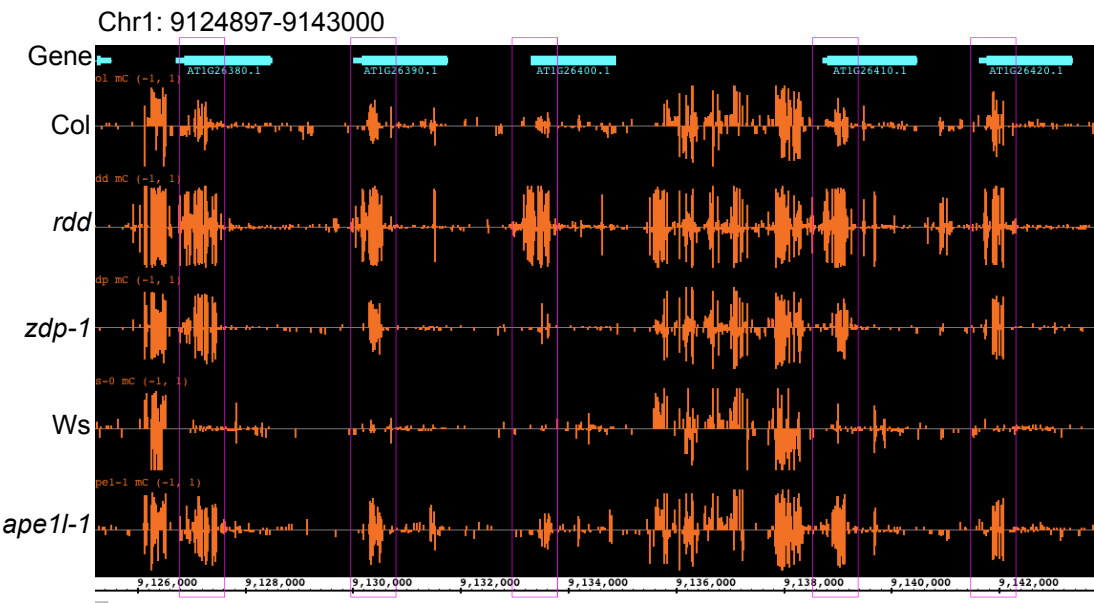

H

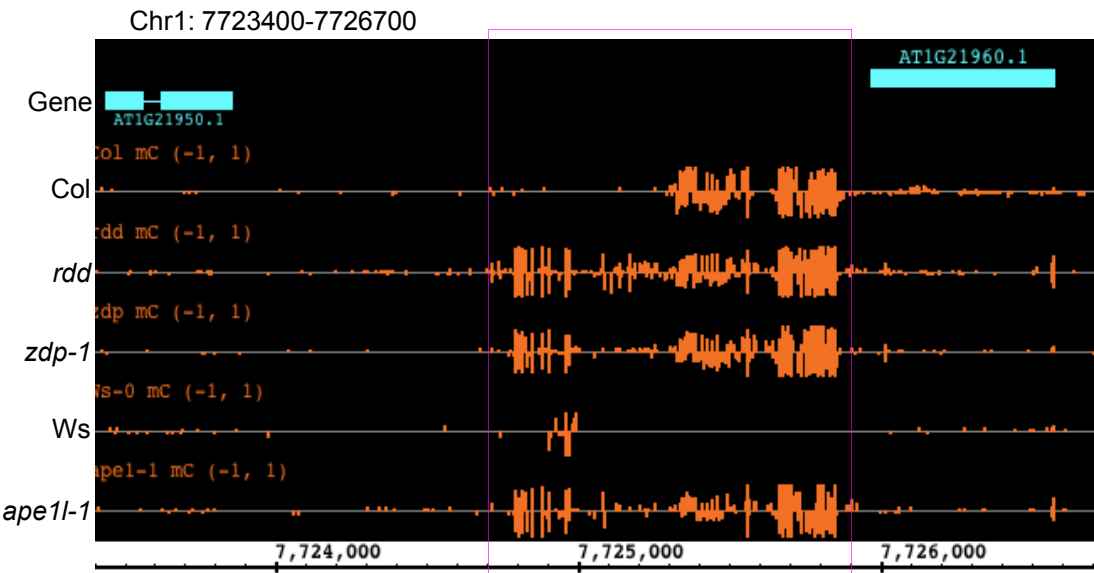

Figure S5 continued

I

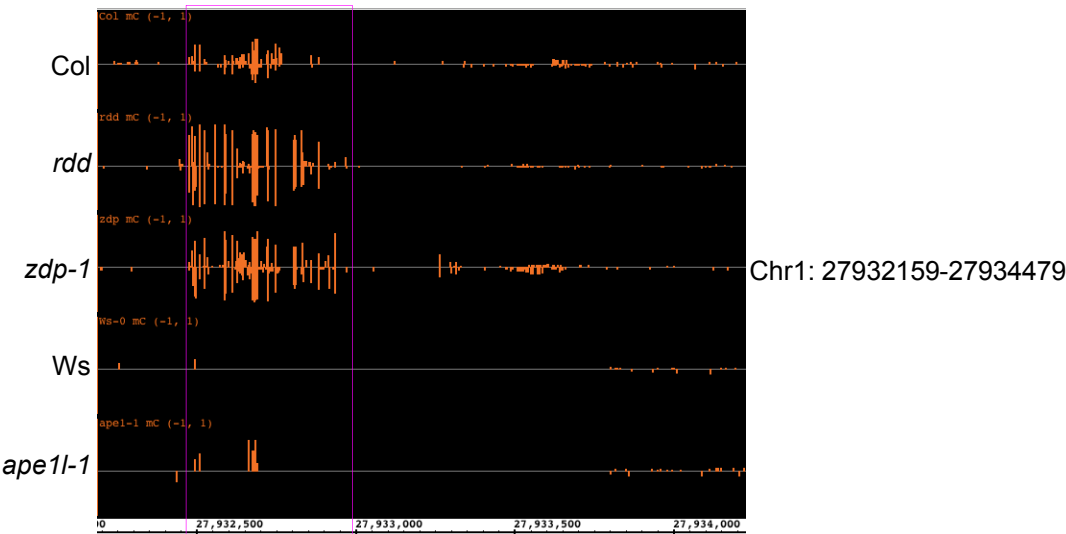

J

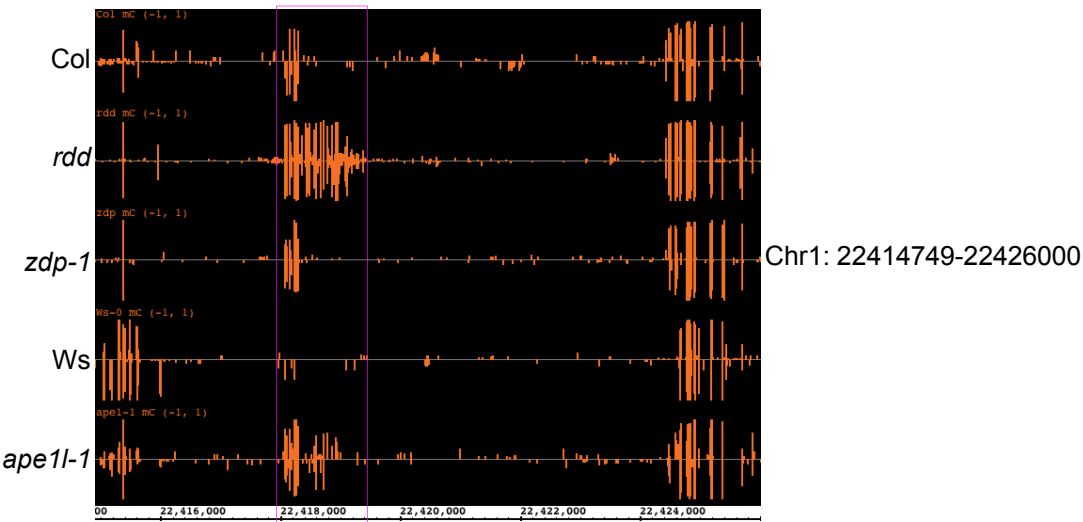

Supplement: S5 Fig — DNA methylome analysis by whole genome bisulfite sequencing in wild-type and ape1l-1 mutant plants. (A) Average methylation levels in gene and TE bodies. Genes or TEs were aligned from 2 kb upstream of transcription start sites to 2 kb downstream of transcription termination sites. (B) Average methylation levels in CG, CHG, or CHH context in genes of different lengths. (C) Average methylation levels in CG, CHG, or CHH context in TEs of different lengths. Gray-ape1l-1, green-Ws, blue-zdp-1 and purple-Col. (D–F) Numbers of hypermethylated regions that are overlapping among or unique to the ape1l-1, zdp-1, ros1-4 and rdd mutants. (G–J) Examples of whole genome bisulfite sequencing data showing DNA hypermethylation in rdd and/or zdp-1 and ape1l-1 mutant plants. Red box-highlighted are regions that are hypermethylated in at least one of the mutants. (PDF) [file pgen.1004905.s005.pdf]

Figure S6

A

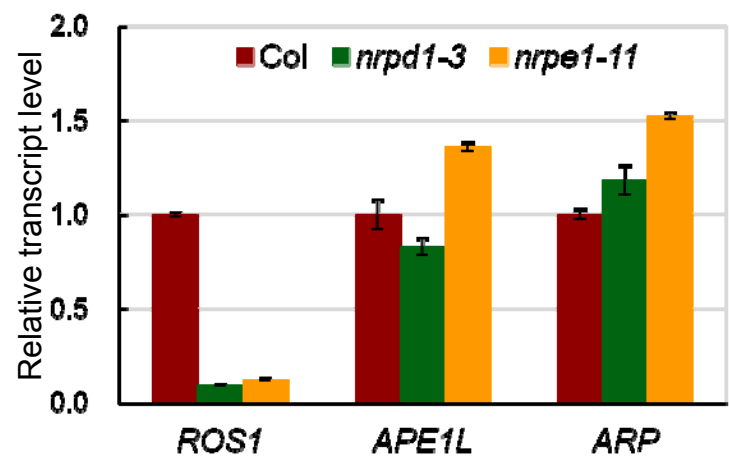

B

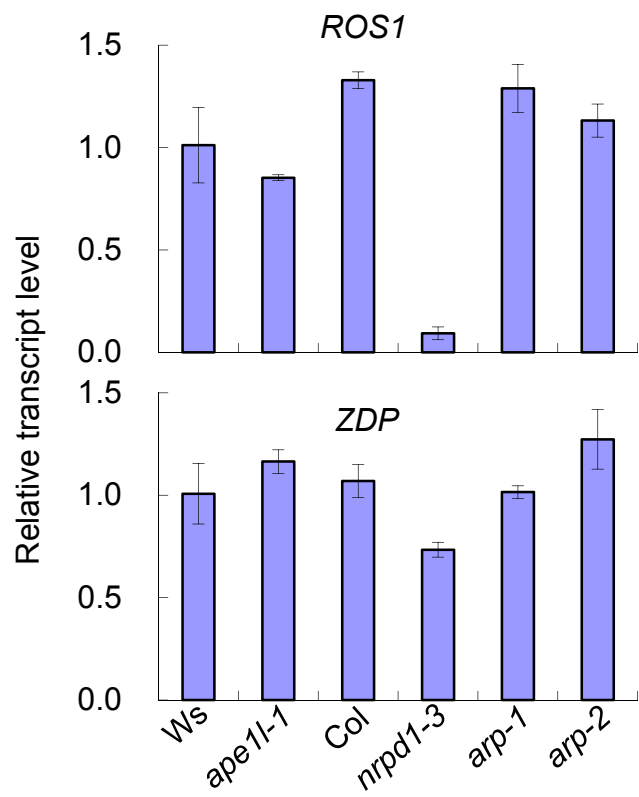

Supplement: S6 Fig — Expression analysis by qRT-PCR. (A) Expression of ROS1, APE1L and ARP in RdDM pathway mutants. (B) Expression of ROS1 and ZDP in the ape and nrpd1-3 mutants. The transcript levels were normalized against ACT 11 expression. Error bars represent standard error (n = 3). (PDF) [file pgen.1004905.s006.pdf]

Figure S7

A

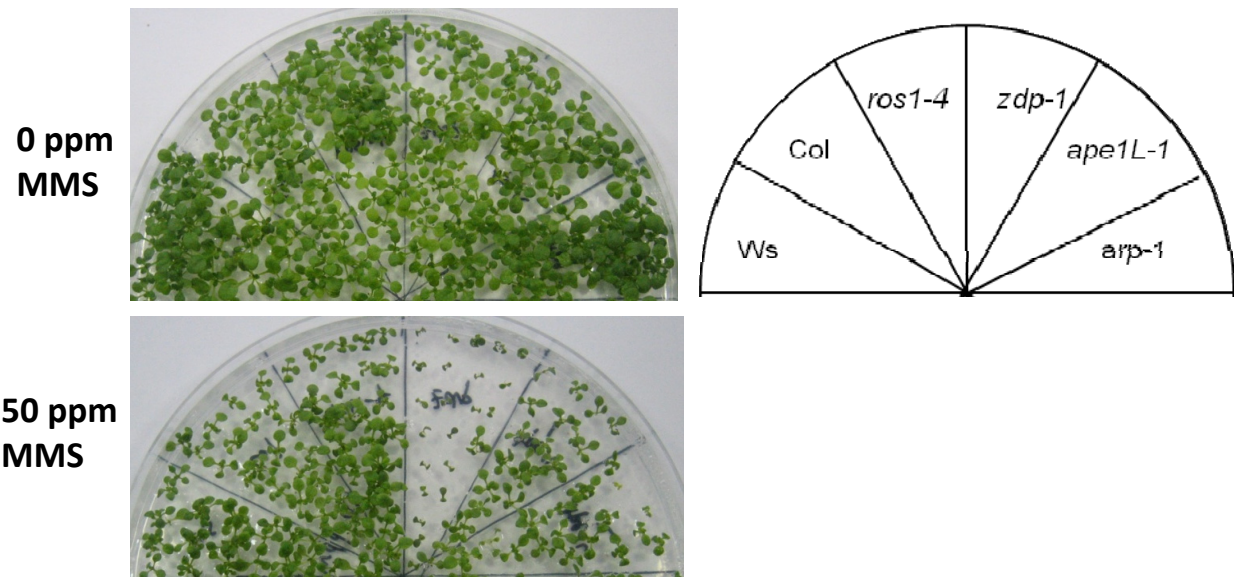

B

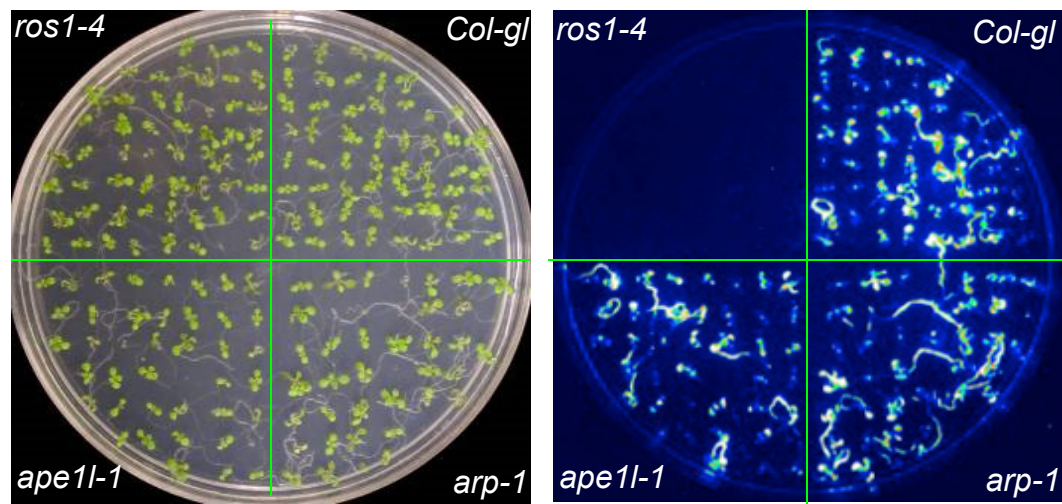

Supplement: S7 Fig — The sensitivity of ros1-4, zdp and ape mutants to MMS and the effect of ape mutant on the RD29A-LUC reporter gene. (A) Plants were grown for 14 days in MS nutrient agar plates containing 0 or 50 ppm MMS. (B) Effect of ape1l-1 and arp-1 on RD29A-LUC reporter gene. The reporter gene was introduced to ape1l-1 and arp-1 mutant plants by crossing. Seedlings grown in MS plate were imaged after cold treatment at 4°C for 24 hours. (PDF) [file pgen.1004905.s007.pdf]

Figure S8

A

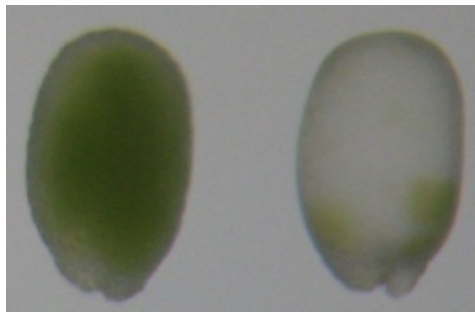

B

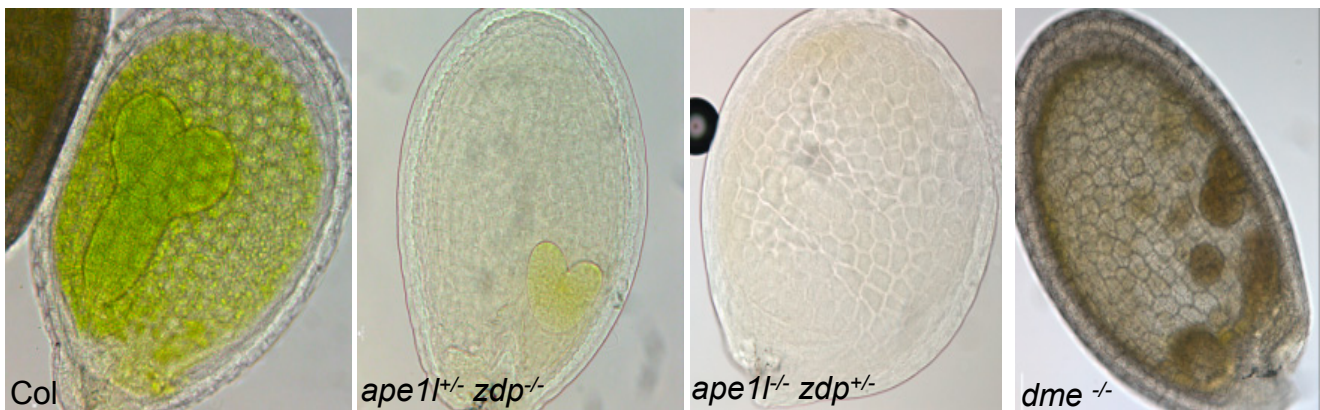

C

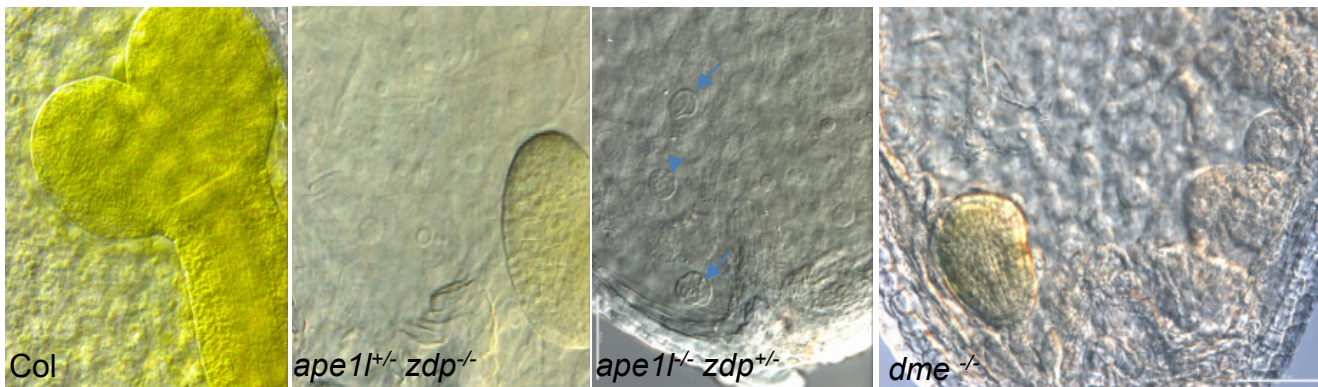

Supplement: S8 Fig — Effects of ape1l and zdp double mutations on seed development. (A) A wild type seed, and an aborting seed showing endosperm over-proliferation (B) Cleared seed samples showing arrested embryo or invisible embryo in ape1l+/−zdp−/−, ape1l−/−zdp+/− and dme+/− mutants as compared to seeds in Col. Images were captured at 8 DAP. (C) Images of cleared seed samples showing unequal sizes of endosperm nuclei in ape1l+/−zdp−/− and ape1l−/−zdp+/− mutants but not in Col and dme+/−. Arrows indicate abnormally large endosperm nuclei. Images were captured at 8 DAP under a differential interference contrast microscope. (PDF) [file pgen.1004905.s008.pdf]

Figure S9

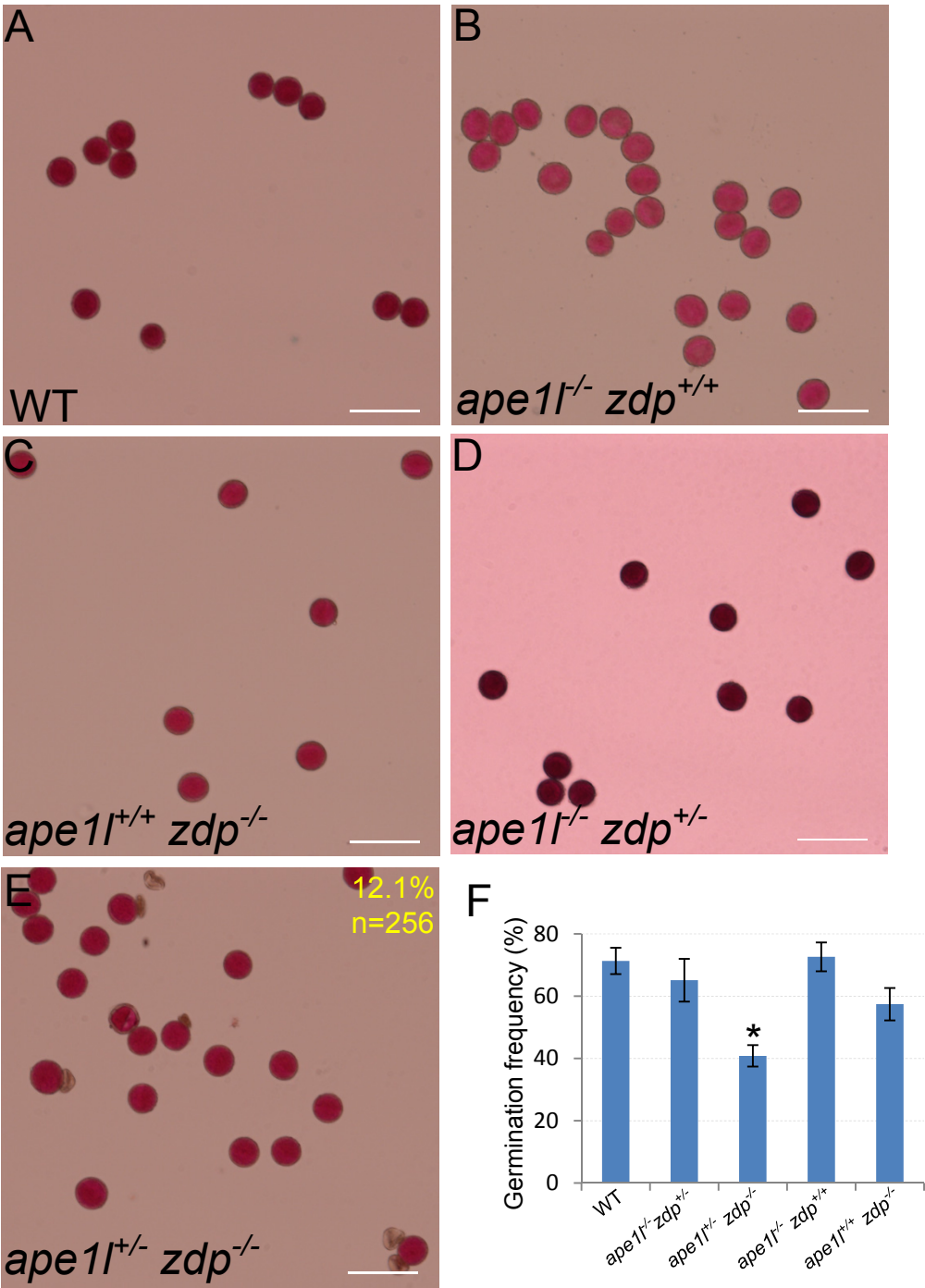

Supplement: S9 Fig — Effects of ape1l and zdp double mutations on pollen development. (A–E) Alexander staining of pollen grains from different genotypes. Viable pollen grains are stained in red. Nonviable pollen grains do not stain red and have different sizes and morphology compared with the viable grains. Bars = 100 µm. (F) In vitro germination of pollen from different genotypes. (*P<0.05). (PDF) [file pgen.1004905.s009.pdf]
