# Supplementary material for: An AP Endonuclease Functions in Active DNA Demethylation and Gene Imprinting in Arabidopsis
Source: PLoS Genet. 2015 Jan 8;11(1):e1004905. doi: 10.1371/journal.pgen.1004905 (PMC4287435; doi:10.1371/journal.pgen.1004905)

Figure S4

A

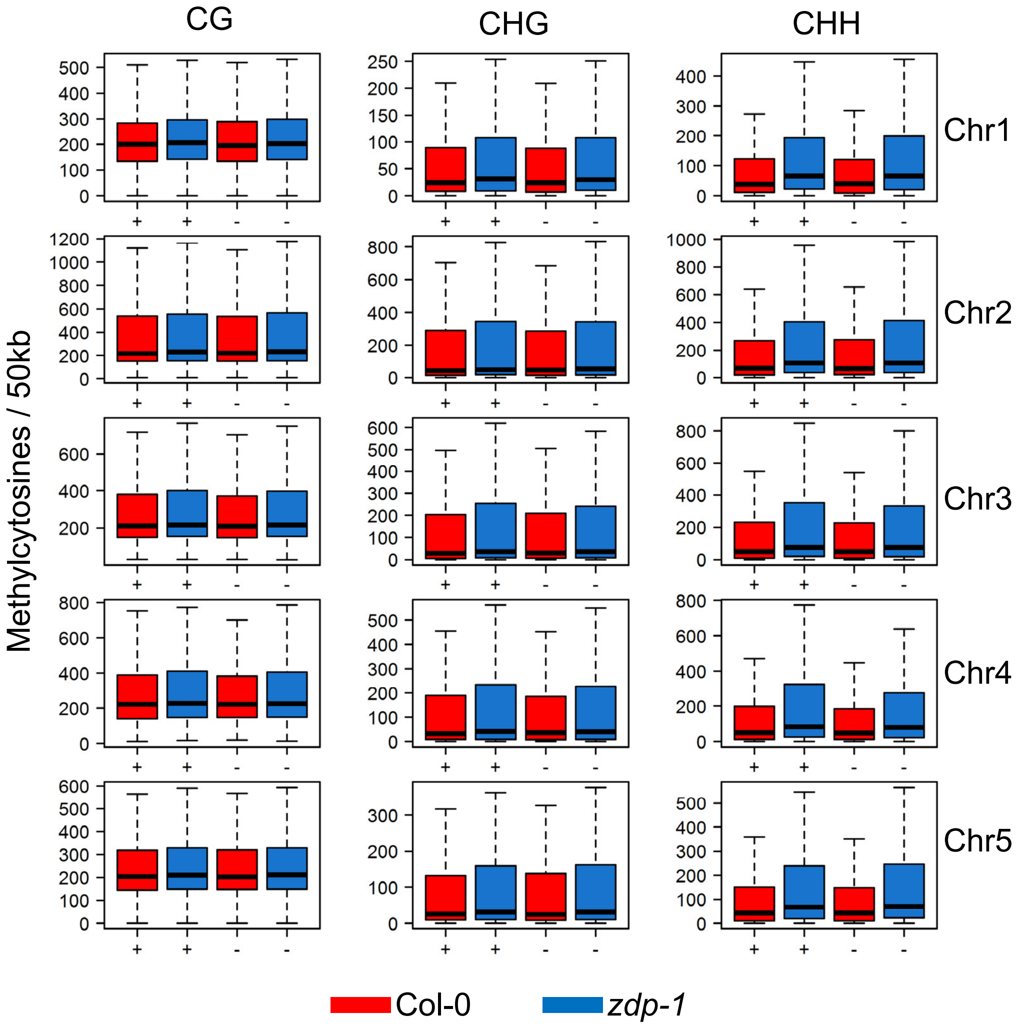

CG
CHG
CHH
Chr1
Chr2
Chr3
Chr4
Chr5
Methylcytosines / 50kb
Col-0
zdp-1

Figure S4 continued

B

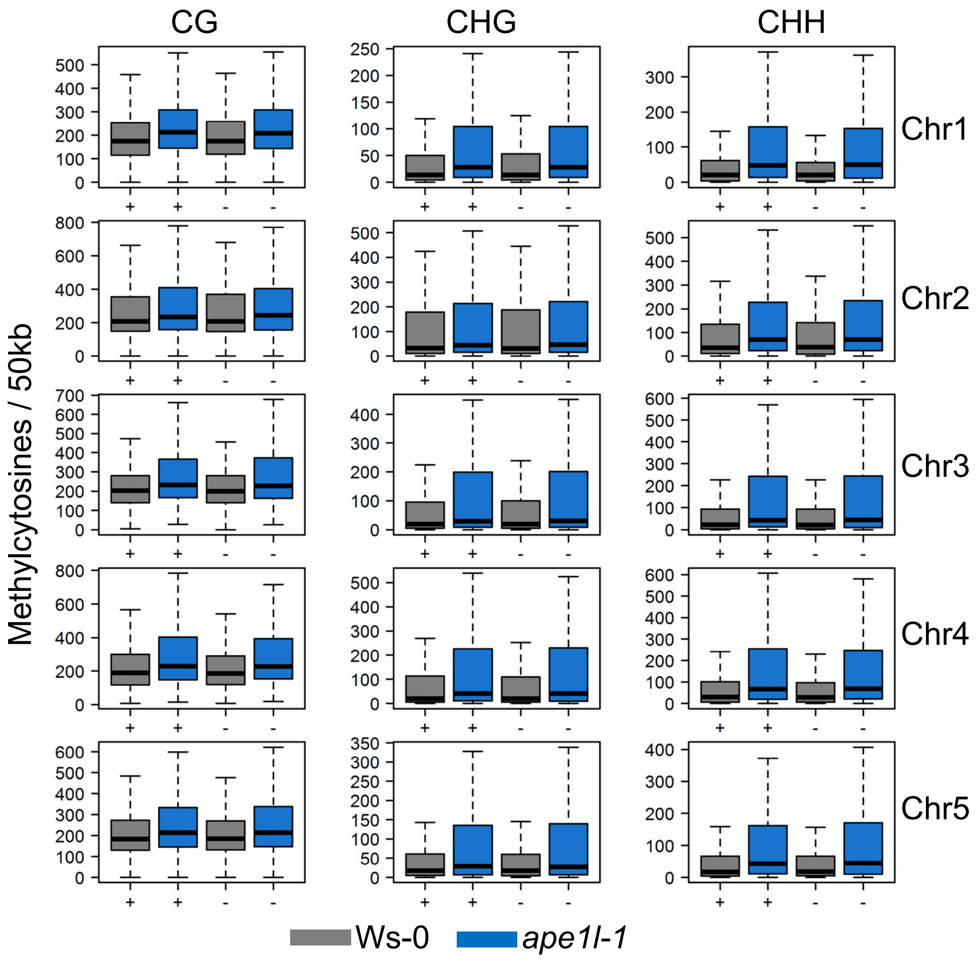

C

Sample	Hyper-DMRs	Hypo-DMRs	Overlap with *rdd*
rdd	9967	991	
ros1-4	6622	646	74.7%
zdp-1	1559	612	70.9%
ape1l-1	3,497	2,892	46.7%
arp-1	162	241	80.2%

Figure S4 continued

D

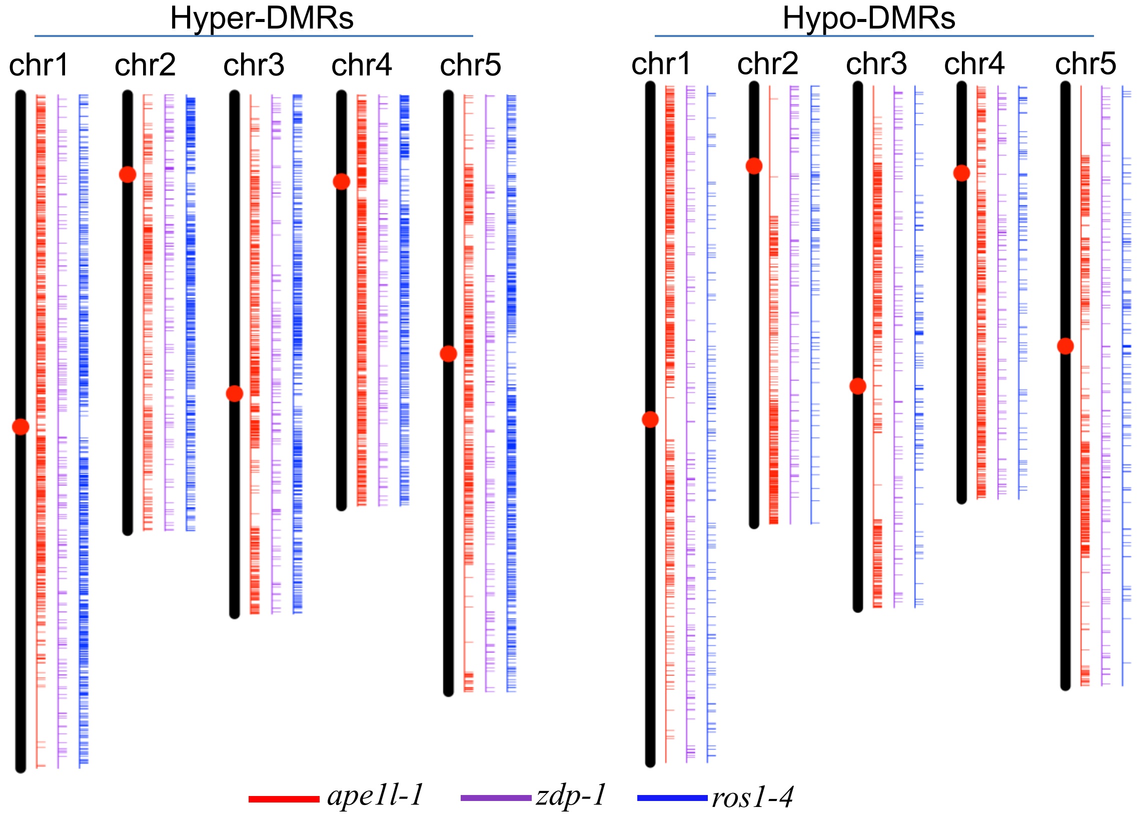

E

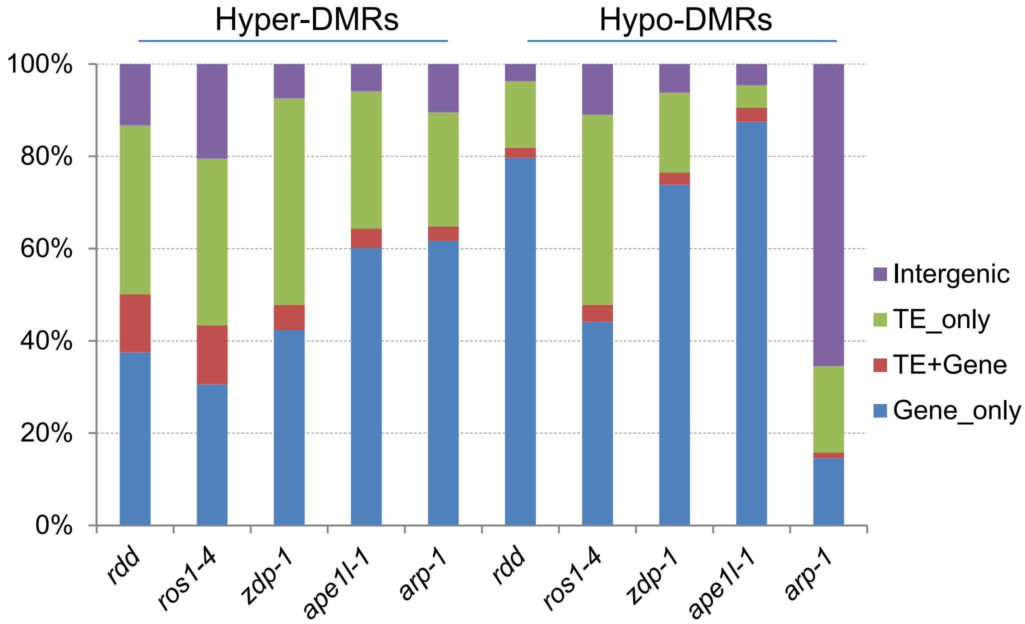

Supplement: S4 Fig — DNA methylome analysis by whole genome bisulfite sequencing in the different mutants. (A–B) Density of DNA methylation in zdp-1 and ape1l-1 mutants as compared to wild type plants. The densities of methylcytosines in each sequence context (CG, CHG, and CHH) across each chromosome in 50 kb segments are shown. (C) Numbers of hyper-DMRs and hypo-DMRs in the mutants examined. The percentages of hypermethylated regions that overlap with those in rdd mutant are shown on the right. (D) Distribution of hyper- and hypo-DMRs on the five chromosomes in the ape1l-1, zdp-1 and ros1-4 mutants. (E) Composition of the hypermethylated and hypomethylated genomic regions in the mutants examined. (PDF) [file pgen.1004905.s004.pdf]
