# Supplementary material for: An AP Endonuclease Functions in Active DNA Demethylation and Gene Imprinting in Arabidopsis
Source: PLoS Genet. 2015 Jan 8;11(1):e1004905. doi: 10.1371/journal.pgen.1004905 (PMC4287435; doi:10.1371/journal.pgen.1004905)
Supplement: S1 Table — Primers and probes used in this study. (DOCX) [file pgen.1004905.s010.docx]

**Table S1.** Primers and probes used in this study.

| Primer name | Sequence 5´ to 3´ | References |
| --- | --- | --- |
| *ape1l-1*-RP | TATGGTTCTGCCATTTCCTTG | *ape1l-1* genotyping |
| *ape1l-1*-LP | GTCGATCCTCATCCTCTTTCC |  |
| Flag line LB4 | CGTGTGCCAGGTGCCCACGGAATAGT |  |
| *ape1l-2*-RP | AAGCACATTTACCCACATTGC | *ape1l-2* genotyping |
| *ape1l-2*-LP | GGCCATCTACAACTTAGCAGC |  |
| Salk line LBa1 | TGGTTCACGTAGTGGGCCATCG |  |
| *arp-1*-RP | CGAGAAAAAGGCATGAACTTG | *arp-1* genotyping |
| *arp-1*-LP | GCTGCAGGACCAGAAACTATG |  |
| *arp-2*-RP | CCAGGAGCAGCTATTGATCAG | *arp-2* genotyping |
| *arp-2*-LP | AGCTTTCCAGTCCTTCTGAGG |  |
| Sail line LB3 | TAGCATCTGAATTTCATAACCAATCTCGATACA |  |
| *ZDP*-RP | AATGAATCCAACATTGATCGATGGAAG | *zdp-1* genotyping |
| *ZDP*-LP | ATACAGCTAAGTCCCTGGCGATGTACTT |  |
| *DME*-5F | GACTCTACGGGAACACATGGA | *dme*  genotyping |
| *DME*-5R | GCTCGTACCTGCAAATAGACG |  |
| L4 | TGATCCATGTAGATTTCCCGGACATGAAG |  |
| *APE1L*-F | TCGGATCCATGAAGCGATTCTTCAAGCCCA | *APE1L* RT-PCR |
| *APE1L*-R | TCGTCGACGTTTGACACCTGGTTCTGTTC |  |
| *ARP*-F | TCGGATCCATGAACAACGTTCTTCAGTTTG | *ARP* RT-PCR |
| *ARP*-R | TCGTCGACGAGCTTGAGAATAAGGCCAA |  |
| *FWA*TkF2 | GTGACTCTGGTCAAGACT | q-PCR using endosperm RNA |
| *FWA*6135r | TTGGTTCCACCAGAACCGGTA |  |
| *MEA*-F | ATTGTGGTCTCAGATCCAAAC |  |
| *MEA*-R | TCTAGGCACGTCTTAAGCC |  |
| *DME*-RTf | CAGAAGTGTGGAGGGAAAGCGTCTGGC |  |
| *DME*-RTr4 | AAATATGTCCCGTTGAGCGGAA |  |
| *FIE*-RTf2 | TACCCGGTTCCAATGTGTGAT |  |
| *FIE*-RTr2 | ATCCCAGACATAAACCTTTCCTTCCT |  |
| *ACT11*-F | GGAACAGTGTGACTCACACCATC |  |
| *ACT11*-R | AAGCTGTTCTTTCCCTCTACGC |  |
| *proFWA-F* | AAACAACAAAAATCTGATTGTCAGTATC | *McrBC* assay |
| *proFWA-R* | ATTCATACGAGCACCGCTTTACG |  |
| *proFIS2-F* | GTTGATTACCAAACCCGAAGAAGA |  |
| *proFIS2-R* | GATTTACTTTATGATTTCGCAGCGG |  |
| APE1LN212D_F4 | ATGGTGTGGCGACTTAGATGTCAGTCATGAGGAGATAG | site-directed mutagenesis |
| APE1LN212D_R4 | CTATCTCCTCATGACTGACATCTAAGTCGCCACACCAT |  |
| *APE1L*-Cluc-F | AATGGATCCATGAAGCGATTCTTCAAGCCCA | *APE1L* construct |
| *APE1L*-Cluc-R | TAAGTCGACTTAGTTTGACACCTGGTTCTG |  |
