# Supplementary material for: An AP Endonuclease Functions in Active DNA Demethylation and Gene Imprinting in Arabidopsis
Source: PLoS Genet. 2015 Jan 8;11(1):e1004905. doi: 10.1371/journal.pgen.1004905 (PMC4287435; doi:10.1371/journal.pgen.1004905)
Supplement: S2 Table — DNA sequence of oligonucleotides used as substrates. (DOCX) [file pgen.1004905.s011.docx]

**Table S2.** DNA sequence of oligonucleotides used as substrates.

| **Name** | **DNA sequence 5´ to 3´ ^a^** | **Strand** | **Label^b^** |
| --- | --- | --- | --- |
| Al-28P | TCACGGGATCAATGTGTTCTTTCAGCTC | Upper | Al at 5´ |
| P30_51 | GGTCACGCTGACCAGGAATACC | Lower | - |
| CGR | AGTGCCCTAGTTACACAAGAAAGTCGAG**G**CCAGTGCGACTGGTCCTTATGG | Lower | - |
| Fl-APGF | TCACGGGATCAATGTGTTCTTTCAGCTC**F**GGTCACGCTGACCAGGAATACC | Upper | Fl at 5´ |
| Al-MGF | TCACGGGATCAATGTGTTCTTTCAGCTC**M**GGTCACGCTGACCAGGAATACC | Upper | Al at 5´ |

^a^F = AP site analog (tetrahydrofuran), M = 5meC ^b^Fl = fluorescein; Al = alexa fluor 647
