# Supplementary material for: An AP Endonuclease Functions in Active DNA Demethylation and Gene Imprinting in Arabidopsis
Source: PLoS Genet. 2015 Jan 8;11(1):e1004905. doi: 10.1371/journal.pgen.1004905 (PMC4287435; doi:10.1371/journal.pgen.1004905)
Supplement: S5 Table — Seeds phenotype in self and reciprocal crosses between ape1l−/−zdp+/−, ape1l+/−zdp−/− and Col. (DOCX) [file pgen.1004905.s014.docx]

**Table S5. Seeds phenotype in self and reciprocal crosses between *ape1l^-/-^zdp^+/-^, ape1l^+/-^zdp^-/-^* and Col.**

| Parental genotype | | number | | | Percentage | | expected | Chi-square | Confidence |
| --- | --- | --- | --- | --- | --- | --- | --- | --- | --- |
| ♀ | ♂ | total | WT | Aborted | WT | Aborted |  |  |  |
| ***ape1l^+/-^***  ***zdp^-/-^*** | ***ape1l^+/-^***  ***zdp^-/-^*** | **497** | **255** | **242** | **51.3%** | **48.7%** | **1:1** | **0.290** | **>0.05** |
| ***ape1l^-/-^***  ***zdp^+/-^*** | ***ape1l^-/-^***  ***zdp^+/-^*** | **868** | **638** | **230** | **73.5%** | **26.5%** | **3:1** | **1.08** | **>0.05** |
| ***ape1l^+/-^***  ***zdp^-/-^*** | **Col** | **516** | **256** | **260** | **49.6%** | **50.4%** | **1:1** | **0.0174** | **>0.05** |
| **Col** | ***ape1l^+/-^***  ***zdp^-/-^*** | **243** | **243** | **0** | **100%** | **0%** |  |  |  |
| ***ape1l^+/-^***  ***zdp^-/-^*** | ***zdp^-/-^*** | **676** | **348** | **328** | **51.5%** | **48.5%** | **1:1** | **0.534** | **>0.05** |
| ***zdp^-/-^*** | ***ape1l^+/-^***  ***zdp^-/-^*** | **265** | **265** | **0** | **100%** | **0%** |  |  |  |
| ***ape1l^-/-^***  ***zdp^+/-^*** | **Col** | **501** | **491** | **10** | **98.0%** | **2.0%** |  |  |  |
| **Col** | ***ape1l^-/-^***  ***zdp^+/-^*** | **244** | **244** | **0** | **100%** | **0%** |  |  |  |
| ***ape1l^-/-^***  ***zdp^+/-^*** | **ape1l*^-/-^*** | **322** | **312** | **10** | **96.9%** | **3.1%** |  |  |  |
| **ape1l*^-/-^*** | ***ape1l^-/-^***  ***zdp^+/-^*** | **423** | **423** | **0** | **100%** | **0%** |  |  |  |
