# Supplementary material for: An AP Endonuclease Functions in Active DNA Demethylation and Gene Imprinting in Arabidopsis
Source: PLoS Genet. 2015 Jan 8;11(1):e1004905. doi: 10.1371/journal.pgen.1004905 (PMC4287435; doi:10.1371/journal.pgen.1004905)
Supplement: S6 Table — The GFP phenotype in self crosses of ape1l−/−zdp+/−, ape1l+/−zdp−/− and pFWA-GFP. (DOCX) [file pgen.1004905.s015.docx]

**Table S6. The GFP phenotype in self crosses of *ape1l^-/-^zdp^+/-^, ape1l^+/-^zdp^-/-^* and *pFWA-GFP.***

| Parental genotype | | number | | | Percentage | | Expected | Chi-square | Confidence |
| --- | --- | --- | --- | --- | --- | --- | --- | --- | --- |
| ♀ | ♂ | total | GFP+ | GFP- | GFP+ | GFP- |  |  |  |
| ***ape1l^+/-^zdp^-/-^; pFWA-GFP*** | ***ape1l^+/-^zdp^-/-^;***  ***pFWA-GFP*** | **293** | **127** | **166** | **43.30%** | **56.70%** | **1:1** | **4.93** | **<0.05** |
| ***ape1l^-/-^zdp^+/-^***  ***pFWA-GFP*** | ***ape1l^-/-^zdp^+/-^:***  ***pFWA-GFP*** | **266** | **138** | **128** | **51.90%** | **48.10%** | **1:1** | **0.305** | **>0.05** |
| ***pFWA-GFP*** | ***pFWA-GFP*** | **351** | **351** | **0** | **100%** | **0%** |  |  |  |
