# Supplementary material for: Correction: An AP Endonuclease Functions in Active DNA Demethylation and Gene Imprinting in Arabidopsis
Source: PLoS Genet. 2015 Apr 27;11(4):e1005198. doi: 10.1371/journal.pgen.1005198 (PMC4411094; doi:10.1371/journal.pgen.1005198)
Supplement: S1 File — (PDF) [file pgen.1005198.s001.pdf]

OPEN ACCESS Freely available online

PLOS | GENETICS

An AP Endonuclease Functions in Active DNA Dimethylation and Gene Imprinting in *Arabidopsis*

Yan Li[1,2‡], Dolores Córdoba-Cañero[3‡], Weiqiang Qian[2‡], Xiaohong Zhu[4], Kai Tang[4], Huiming Zhang[1], Rafael R. Ariza[3], Teresa Roldán-Arjona[3]*, Jian-Kang Zhu[1,4]*

1 Shanghai Center for Plant Stress Biology, Shanghai Institutes for Biological Sciences, Chinese Academy of Sciences, Shanghai, China, **2** State Key Laboratory of Protein and Plant Gene Research, School of Life Sciences and Peking-Tsinghua Center for Life Science, Peking University, Beijing, China, **3** Department of Genetics, University of Córdoba/Maimonides Institute for Biomedical Research of Cordoba (IMIBIC)/Reina Sofía University Hospital, Córdoba, Spain, **4** Department of Horticulture & Landscape Architecture, Purdue University, West Lafayette, Indiana, United States of America

Abstract

Active DNA demethylation in plants occurs through base excision repair, beginning with removal of methylated cytosine by the ROS1/DME subfamily of 5-methylcytosine DNA glycosylases. Active DNA demethylation in animals requires the DNA glycosylase TDG or MBD4, which functions after oxidation or deamination of 5-methylcytosine, respectively. However, little is known about the steps following DNA glycosylase action in the active DNA demethylation pathways in plants and animals. We show here that the *Arabidopsis* APE1L protein has apurinic/apyrimidinic endonuclease activities and functions downstream of ROS1 and DME. APE1L and ROS1 interact *in vitro* and co-localize *in vivo*. Whole genome bisulfite sequencing of *ape1l* mutant plants revealed widespread alterations in DNA methylation. We show that the *ape1l/zdp* double mutant displays embryonic lethality. Notably, the *ape1l*$^{+/-}$*zdp*$^{-/-}$ mutant shows a maternal-effect lethality phenotype. APE1L and the DNA phosphatase ZDP are required for *FWA* and *MEA* gene imprinting in the endosperm and are important for seed development. Thus, APE1L is a new component of the active DNA demethylation pathway and, together with ZDP, regulates gene imprinting in *Arabidopsis*.

Citation: Li Y, Córdoba-Cañero D, Qian W, Zhu X, Tang K, et al. (2015) An AP Endonuclease Functions in Active DNA Dimethylation and Gene Imprinting in *Arabidopsis*. PLoS Genet 11(1): e1004905. doi:10.1371/journal.pgen.1004905

Editor: Craig S. Pikaard, Indiana University, Howard Hughes Medical Institute, United States of America

Received July 21, 2014; **Accepted** November 18, 2014; **Published** January 8, 2015

Copyright: © 2015 Li et al. This is an open-access article distributed under the terms of the Creative Commons Attribution License, which permits unrestricted use, distribution, and reproduction in any medium, provided the original author and source are credited.

Data Availability: The authors confirm that all data underlying the findings are fully available without restriction. Whole-genome bisulfite sequencing was used to analyze the methylomes of Ws, ape1l-1, arp-1 and zdp-1 mutant plants. The data set was deposited at NCBI (GSE52983).

Funding: This work was supported by the National Institutes of Health Grants R01GM070795 and R01GM059138 (to JKZ), by the Spanish Ministry of Economy and Competitiveness/European Regional Development Fund Grant BFU2013-43269-P and Junta de Andalucía Grant P11-CVI-7576 (to TRA), and by the Chinese Academy of Sciences and by Peking-Tsinghua Center for Life Science. The funders had no role in study design, data collection and analysis, decision to publish, or preparation of the manuscript.

Competing Interests: The authors have declared that no competing interests exist.

* Email: ge2roarm@uco.es (TRA); jkzhu@purdue.edu (JKZ)

‡ These authors contributed equally to this work.

Introduction

DNA methylation is a stable epigenetic mark that regulates numerous aspects of the genome, including transposon silencing and gene expression [1–7]. In plants, DNA methylation can occur within CG, CHG, and CHH motifs (H represents A, T, or C). Genome-wide mapping of DNA methylation in *Arabidopsis* has revealed that methylation in gene bodies is predominantly at CG context whereas methylation in transposon- and other repeat-enriched heterochromatin regions can be within all three motifs [8]. Although the function of abundant CG methylation within genic regions remains unclear, DNA methylation generally correlates with histone modifications that repress transcription activities [1,9,10]. DNA methylation patterns are coordinately controlled by methylation and demethylation reactions. In *Arabidopsis*, symmetric CG and CHG methylation can be maintained by DNA METHYLTRANSFERASE 1 (MET1) and CHROMOMETHYLASE 3 (CMT3), respectively, during DNA replication. In contrast, asymmetric CHH methylation cannot be maintained and is established *de novo* by DOMAINS REARRANGED METHYLASE 2 (DRM2), which can be targeted to specific sequences by the RNA-directed DNA methylation (RdDM) pathway [1,10,11]. DNA methylation is antagonized by an active DNA demethylation pathway that includes the DNA glycosylases REPRESSOR OF SILENCING1 (ROS1), DEMETER (DME), DEMETER-LIKE2 (DML2) and DEMETER-LIKE3 (DML3) [12–14].

ROS1, DME, DML2 and DML3 are all bifunctional DNA glycosylases that initiate active DNA demethylation by removing the 5-methylcytosine (5-meC) base and subsequently cleaving the phosphodiester backbone by either β- or β, δ-elimination [12,14–16]. When β, δ-elimination occurs, a gap with a 3′-phosphate group is generated. Our previous work demonstrated that the 3′ DNA phosphatase ZDP catalyzes the conversion of 3′-phosphate group to a 3′-hydroxyl (3′-OH), enabling DNA polymerase and ligase activities to fill in the gap [17]. The β-elimination product, a gap with a blocking 3′-phosphor-α, β-unsaturated aldehyde (3′-PUA), also must be converted to a 3′-OH to allow completion of the demethylation process through single-nucleotide insertion or long patch DNA synthesis by DNA

Author Summary

DNA cytosine methylation (5-methylcytosine, 5-meC) is an important epigenetic mark, and methylation patterns are coordinately controlled by methylation and demethylation reactions during development and reproduction. In plants, REPRESSOR OF SILENCING (ROS1) is one of the well characterized 5-meC DNA glycosylases that initiate active DNA demethylation by 5-meC excision. Our previous work showed that a 3′-DNA phosphatase, ZDP, functions downstream of ROS1 during active DNA demethylation in *Arabidopsis*. Here we found that the apurinic/apyrimidinic endonuclease APE1L functions downstream of ROS1 in a ZDP-independent branch of the active DNA demethylation pathway in *Arabidopsis*. In plants, gene imprinting requires the 5-meC DNA glycosylase Demeter (DME) that has been proposed to initiate a base excision repair pathway for active DNA demethylation in the central cell in female gametophyte. However, besides DME, no other base excision repair enzymes have been found to be important for gene imprinting. Our results show that APE1L and ZDP act jointly downstream of DME to regulate gene imprinting in plants, and suggest that DME-initiated active DNA demethylation in the central cell and endosperm uses both APE- and ZDP-dependent mechanisms.

polymerase and ligase [18]. However, the enzymes that may function downstream of ROS1 and DME in the β-elimination pathway have not been identified.

The mutation of *ROS1* leads to hypermethylation and transcriptional silencing of a luciferase reporter gene driven by the *RD29A* promoter, as well as of the endogenous *RD29A* gene [13]. *ROS1* dysfunction also causes DNA hypermethylation in thousands of endogenous genomic regions [19]. *zdp* mutants also show hypermethylation in the *RD29A* promoter and many endogenous loci. However, the hypermethylation in the *RD29A* promoter caused by *zdp* mutations is not as high as that caused by *ros1* mutations, and there are many ROS1 targets that are not hypermethylated in *zdp* mutants [17]. These observations suggest that there may be an alternative, ZDP-independent branch of the DNA demethylation pathway downstream of ROS1 and other DNA glycosylases/lyases.

Although ROS1 functions in almost all plant tissues [13], DME is preferentially expressed in the central cell of the female gametophyte and is important for the regulation of gene imprinting in the endosperm [20–22]. In *Arabidopsis*, the imprinted protein-coding genes include *FWA* (Flowering Wageningen), *MEA* (MEDEA) and *FIS2* (Fertilization-Independent Seed 2) and the list is expanding [21–25]. The loss-of-function mutation of *DME* results in aberrant endosperm and embryo development because of DNA hypermethylation and down-regulation of the maternal alleles of imprinted genes [26]. DME is also necessary for DNA demethylation in the companion cells in the male gametophyte [27–29]. SSRP1, a chromatin remodeling protein, was identified as another factor required for gene imprinting and the mutation of *SSRP1* gives rise to a maternal lethality phenotype similar to that caused by *DME* mutations [30]. Therefore, it is possible that ZDP and other protein(s) acting downstream of the 5-meC DNA glycosylases/lyases may also affect gene imprinting in *Arabidopsis*. Intriguingly though, neither ZDP mutants nor mutants in other DNA repair enzymes that may be downstream of DNA glycosylases/lyases show developmental phenotypes associated with defective gene imprinting.

In this study, we characterized the functions of *Arabidopsis* APE-like proteins in the processing of 3′-blocking ends generated by ROS1 and examined methylome changes induced by *ape* mutations. We found that purified APE1L can process 3′-PUA termini to generate 3′-OH ends. APE1L also displays a weak activity in converting 3′-phosphate termini to 3′-OH ends. *ape1l-1* mutants show altered methylation patterns in thousands of genomic regions. Interestingly, we found that the $ape1l^{+/-}zdp^{-/-}$ mutant is maternally lethal, giving rise to a seed abortion phenotype resembling that of *dme* mutants. The maternal alleles of the imprinted genes *FWA* and *MEA* are hypermethylated, and their expression levels are reduced in the endosperm of such abnormal seeds of the double mutant. Thus, APE1L functions downstream of the ROS1/DME subfamily of DNA glycosylases/lyases in active DNA demethylation and genomic imprinting in *Arabidopsis*.

Results

APE1L possesses a potent activity against 3′-PUA termini generated by ROS1

The *Arabidopsis* genome encodes three AP endonuclease-like proteins: APE1L, APE2 and ARP [31]. We purified recombinant full-length APE1L, APE2 and ARP proteins, and found that all three enzymes exhibit AP endonuclease activity *in vitro*. APE1L, but not APE2 or ARP, also displayed a 3′-phosphatase activity (Fig. 1A). We next wanted to determine if these proteins can process the 3′-PUA termini generated by ROS1 after the β-elimination reaction. We first incubated ROS1 with a 51-mer duplex DNA substrate containing a 5-meC residue at position 29 in the 5′-end labeled strand (Fig. 1B). As expected, the DNA glycosylase/lyase activity of ROS1 generated a mixture of β- and β, δ -elimination products, with either 3′-PUA or 3′-phosphate ends, respectively (Fig. 1B, lane 1). These products were then purified and combined with either APE1L, APE2 or ARP proteins. We found that APE1L efficiently processed the 3′-PUA to generate a 3′-OH terminus. In comparison, 10-fold higher amounts of APE2 or ARP proteins displayed either weak [32] or undetectable (APE2) activity against 3′-PUA ends (Fig. 1B).

To confirm that APE1L is responsible for the detected enzymatic activity we generated an APE1L mutant, N224D. Residue N224 corresponds to N212 of human APE1, which is essential for the enzymatic activity of the mammalian protein [33]. Substitution of N224 by aspartic acid almost completely abolished the activity of APE1L on the 3′-PUA termini (Fig. 1C). The mutation also greatly reduced the AP endonuclease activity on a synthetic AP site and the 3′ phosphatase activity on 3′-phosphate ends. Altogether these results indicate that, in addition to its AP endonuclease activity, APE1L possesses a potent 3′-phosphodiesterase activity that can efficiently process the 3′-PUA blocking ends generated by ROS1.

APE1L is able to process 3′-PUA and 3′-phosphate termini in the presence of ROS1, but does not increase the enzymatic turnover of ROS1

ROS1 remains bound to its reaction products, which contributes at least partially to the highly distributive behavior of the enzyme *in vitro* [34]. To determine whether APE1L is able to process 3′-PUA and/or 3′-phosphate termini in the presence of ROS1, we incubated ROS1 and a duplex DNA substrate containing a single 5-meC residue, with WT or N224D APE1L (Fig. 2A). We found that a 3′-OH terminus is efficiently generated in the presence of WT but not mutant APE1L (Fig. 2A). The emergence of the 3′-OH terminus is concomitant with the loss of

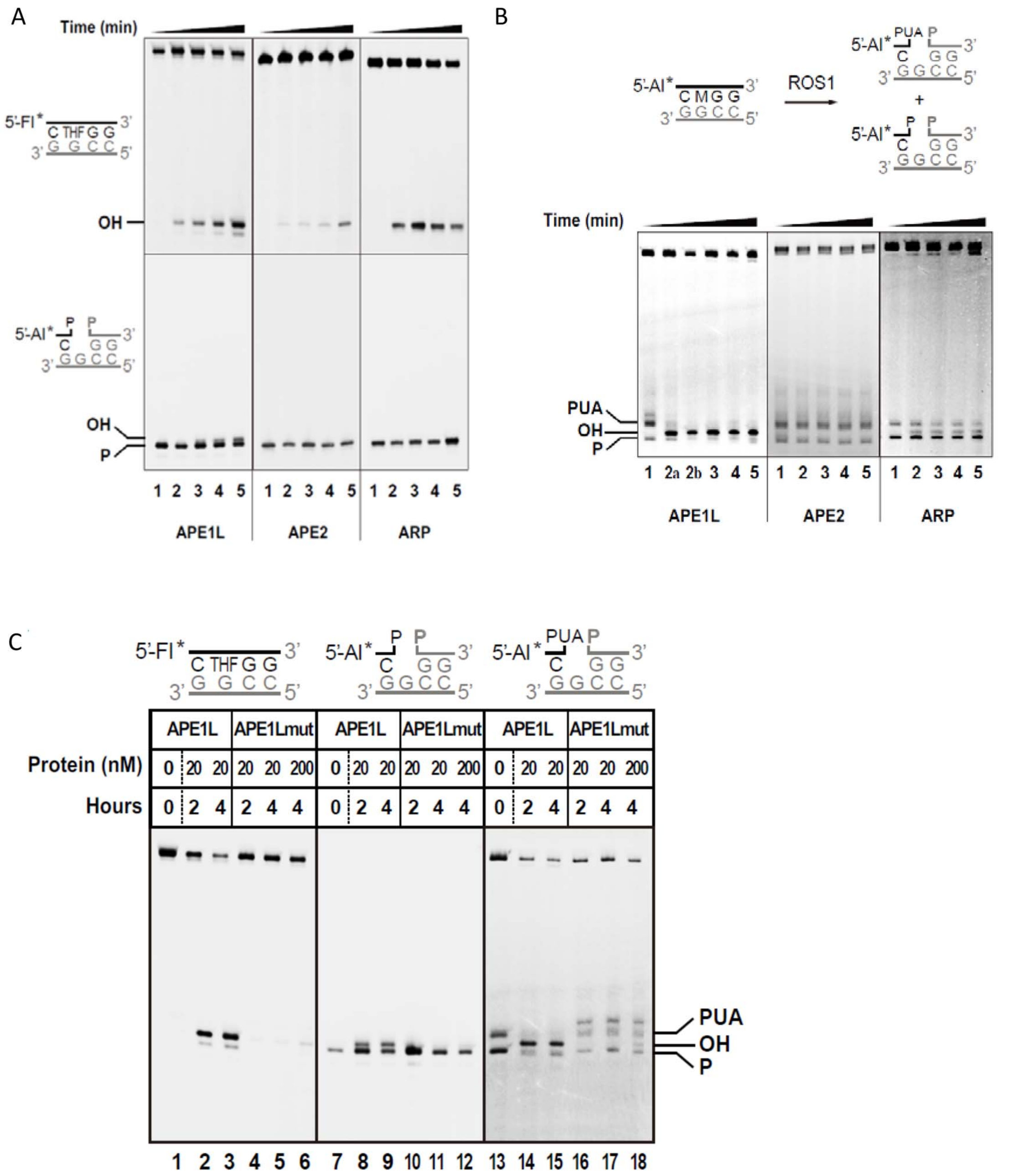

Fig. 1. DNA repair activities of APE1L, APE2 and ARP. (**A**) The AP endonuclease (upper panels) and 3′-phosphatase activities (lower panels) were tested by incubating proteins (20 nM) with DNA duplexes (40 nM) containing either a synthetic AP site (tetrahydrofuran, THF) opposite G, or a single-nucleotide gap flanked by 3′-phosphate and 5′-phosphate termini, respectively. Reactions were stopped at 0, 15, 30, 60 and 120 minutes and products were separated using a 12% denaturing polyacrylamide gel and detected by fluorescence scanning. (**B**) Activity against ROS1 products. Purified His-ROS1 (20 nM) was incubated at 30°C for 4 h with a DNA duplex (20 nM) containing a 5-meC residue opposite G. Reaction products (which contained a mixture of β- and β, δ-elimination products with 3′-PUA and 3′-phosphate ends, respectively, lane 1), were purified and incubated with APE1L, APE2 or ARP (20 nM). Reactions were stopped at 0, 15, 30, 60 and 120 minutes and products were detected as described above. Reaction in lane 2a was performed for 15 min with 2 nM APE1L. (**C**) Mutation N224D drastically reduces the repair activities of APE1L. WT and mutant versions of APE1L (20 nM or 200 nM, as indicated) were incubated with DNA substrates containing, opposite G, either a THF (40 nM), a single-nucleotide gap flanked by 3′-phosphate and 5′-phosphate termini (40 nM), or a mixture of β- and β, δ-elimination products generated by His-ROS1 (20 nM). Reactions were stopped at 2 or 4 h, as indicated, and products were separated using a 12% denaturing polyacrylamide gel and detected by fluorescence scanning.

doi:10.1371/journal.pgen.1004905.g001

both 3′-PUA and 3′-phosphate ends, suggesting that the 3′-OH terminus is produced by the 3′-phosphodiesterase activity of APE1L on the 3′-blocking ends generated by ROS1. Quantification of the reaction products revealed that the total amount of strand incision is not increased in the presence of APE1L (Fig. 2B).

To assess whether APE1L modulates the DNA glycosylase/lyase activity of ROS1, we performed the reaction in the absence of Mg^{2+}, which is required for APE1L but not ROS1 activity. We found that the enzymatic activity of ROS1 is not increased in the presence of APE1L (S1 Fig.). Thus, APE1L is able to access the

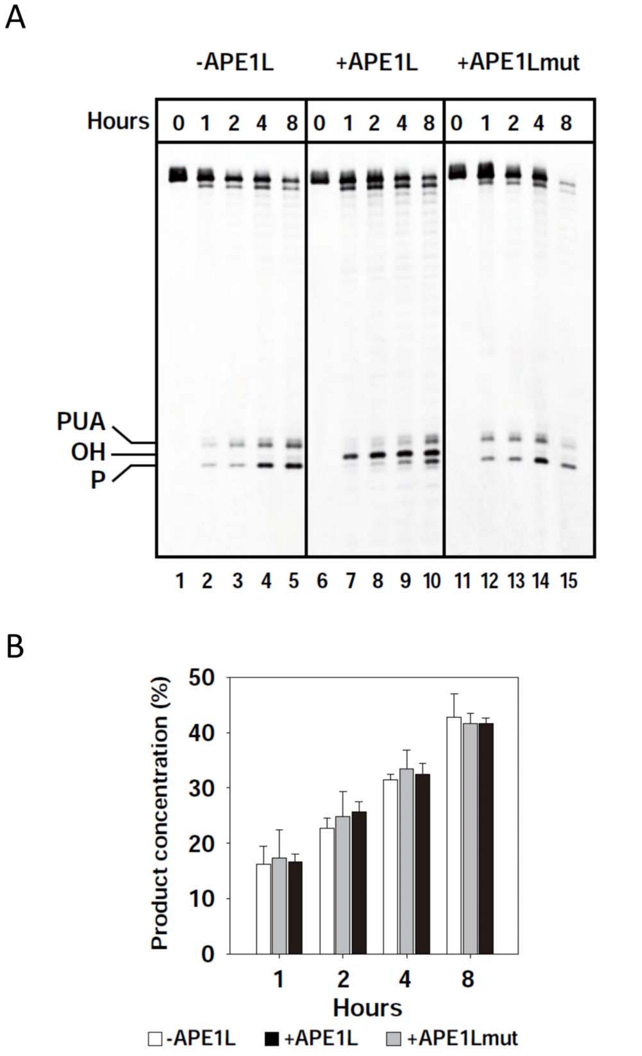

Fig. 2. APE1L is able to process 3′-PUA and 3′-phosphate in the presence of ROS1. (**A**) His-ROS1 (20 nM) was incubated with a DNA substrate containing a 5-meC residue opposite G (20 nM) either in the absence (left panel) or the presence of WT APE1L (2 nM, center panel) or APE1Lmut (20 nM, right panel). Reactions were stopped at the indicated times and products were separated using a 12% denaturing polyacrylamide gel and detected by fluorescence scanning. (**B**) The total amount of incision products was quantified by fluorescence scanning. Values are means with standard errors from two independent experiments.

doi:10.1371/journal.pgen.1004905.g002

3′-blocked termini generated by ROS1 but does not increase the turnover of this DNA glycosylase. These results suggest that APE1L does not displace ROS1 from DNA.

APE1L and ROS1 interact *in vitro* and *in vivo* and form a ternary complex with a gapped DNA substrate

We next used *in vitro* pull-down assays to test whether ROS1 and APE1L can physically interact (Fig. 3A). His-tagged ROS1 (His-ROS1) was incubated with either Maltose Binding Protein (MBP) or MBP-APE1L bound to an amylose column. We found that MBP-APE1L, but not MBP, associates with His-ROS1, suggesting that APE1L and ROS1 directly interact *in vitro*.

To gain insights into the transfer of DNA demethylation intermediates between ROS1 and APE1L, we performed electrophoretic mobility shift assays with a gapped DNA substrate (Fig. 3B–C). MBP-APE1L alone is not able to form a stable complex with the substrate, judging by the smeared band next to the position of the free probe (Fig. 3B, lanes 2 and 1). A mobility shift was observed when the DNA substrate was incubated with His-ROS1, consistent with complex formation (Fig. 3B, lane 3). As we have previously reported [17], part of the labeled probe remained trapped in the wells, hinting at the formation of insoluble His-ROS1-DNA complexes. Next, we incubated the gapped DNA substrate and His-ROS1 with increasing concentrations of MBP-APE1L to assess complex formation. With increasing MBP-APE1L, the band corresponding to the ROS1-DNA complex and the labeled material in the well gradually disappeared, concomitant with the appearance of a discrete, new band (Fig. 3B, lanes 4–7). Importantly, this band was only detected when both ROS1 and APE1L were present in the binding reaction. These results suggest that ROS1, APE1L, and gapped DNA form a ternary complex and that ROS1 is required for APE1L to stably associate with the DNA substrate.

To further examine complex formation we performed supershift experiments using antibodies against MBP-APE1L and His-ROS1 (Fig. 3C). We found that adding anti-MBP to a binding reaction containing MBP-APE1L, His-ROS1 and DNA generated an additional shift, thus confirming the presence of APE1L in the complex. However, a supershift was not observed in the presence of the anti-His antibody. We reasoned that access to the His epitope on His-ROS1 might be restricted in the complex. Therefore, as an alternative approach, we compared the mobility shifts generated from binding reactions containing the DNA gapped substrate, MBP-APE1L and either His-ROS1 or MPB-ROS1 (Fig. 3D). We found that MBP-ROS1, which has a higher molecular weight than His-ROS1, gave rise to a higher molecular weight gel shift, thus confirming that ROS1 is also present in the complex. The most likely interpretation for these results is that ROS1, APE1L, and the gapped DNA substrate form a ternary complex. To further confirm the interaction between APE1L and ROS1, we performed a firefly luciferase complementation imaging assay [35] in tobacco leaves. We found that APE1L can interact with ROS1 in the tobacco leaves (Fig. 3E).

APE1L co-localizes with ROS1 *in vivo*

Our previous data show that ZDP, a component of the active DNA demethylation pathway, co-localizes with ROS1 in subnuclear foci [17]. To determine the subnuclear localization of APE1L protein, we generated antibodies specific to APE1L and used them for immunolocalization of APE1L in *Arabidopsis* leaf nuclei. As shown in Fig. 4A, APE1L is broadly distributed throughout the nucleus. In 62% of the cells examined, APE1L is enriched in the nucleolus whereas in 38% of the cells, APE1L localizes to small nucleoplasmic foci. Only very weak signals were observed when the antibodies were applied to nuclei preparations of *ape1l-1* mutant plants, indicating that the staining patterns in wild type plants reflect APE1L localization rather than non-specific binding of the antibody (Fig. 4A).

To test whether APE1L co-localizes with ROS1 or ZDP, we performed co-immunofluorescence. In our experiments, FLAG-tagged ROS1 was expressed from its native promoter in *ros1-1* mutants and visualized with anti-FLAG antibodies. We observed APE1L co-localization with ROS1 within both nucleoplasmic foci and also the nucleolus in about 10% of cells, as shown by the strong yellow signals (Fig. 4B). In 54% of the cells, APE1L co-localizes with ROS1 in the nucleolus but not in nucleoplasmic foci, whereas in 36% of the cells, APE1L and ROS1 do not substantially co-localize (Fig. 4B). APE1L and ZDP also

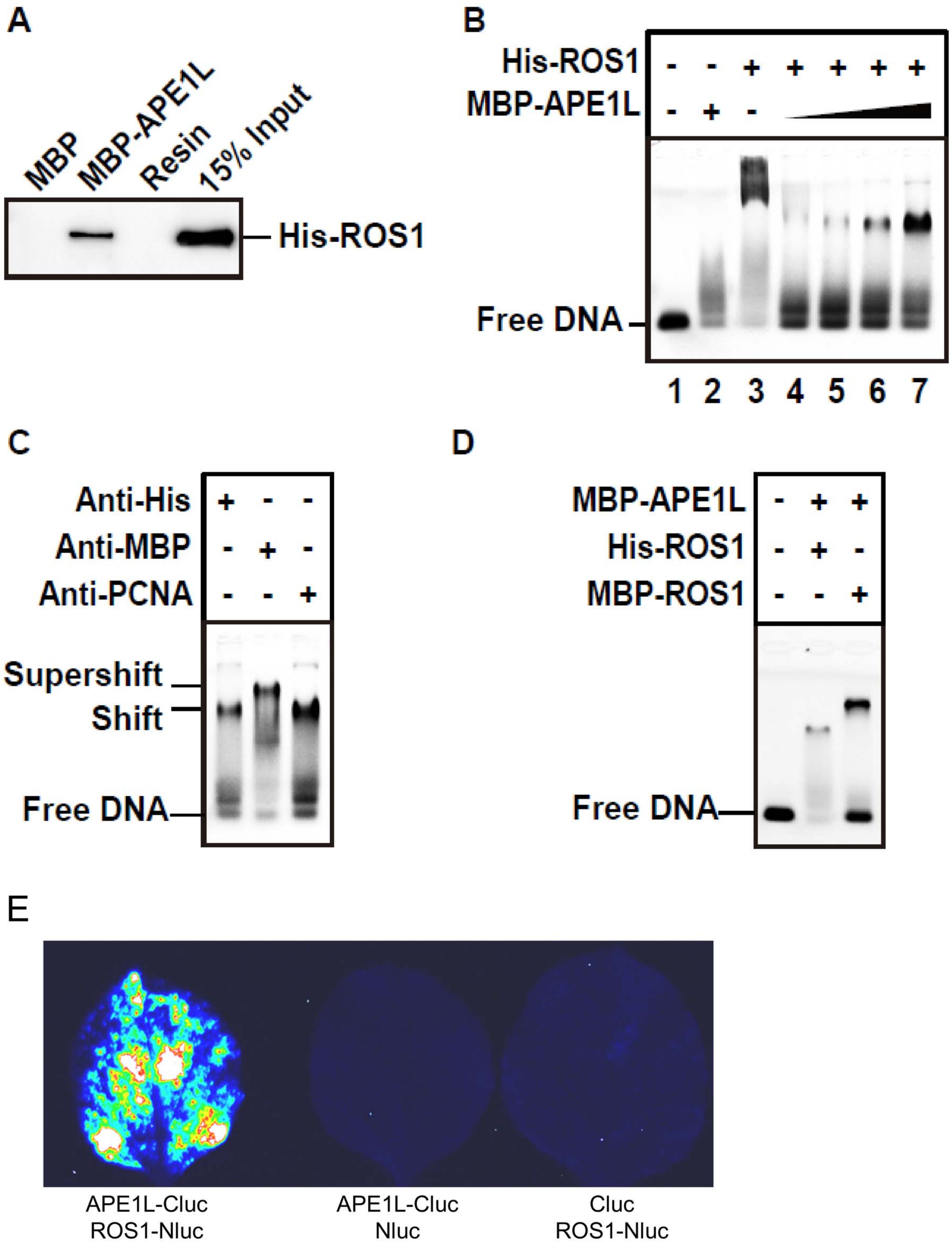

Fig. 3. APE1L and ROS1 interact *in vitro* and in tobacco leaves and form a ternary complex with DNA. (**A**) Pull-down assay. His-ROS1 was incubated with either MBP or MBP-APE1L bound to an amylose column. After washes, the proteins associated to the resin were separated by SDS-PAGE, transferred to a membrane, and immunoblotted with an antibody against the His-tag. (**B**) Electrophoretic mobility shift assay. Purified His-ROS1 (75 nM) and increasing concentrations of MBP-APE1L (0, 250, 500, 750 and 1000 nM) were incubated for 15 min at 25°C with a labeled DNA duplex (10 nM) containing a single-nucleotide gap flanked by 3′-phosphate and 5′-phosphate termini. After non-denaturing gel electrophoresis, protein-DNA complexes were identified by their retarded mobility compared with that of free DNA, as indicated. (**C**) His-ROS1 (75 nM) and MBP-APE1L (1000 nM) were pre-incubated for 4 hours at 15°C with either anti-His or anti-MBP antibodies, and then incubated for 15 min at 25°C with the labeled DNA duplex (10 nM). A control preincubation with anti-PCNA was also performed. Protein-DNA complexes were detected as indicated above. (**D**) MBP-APE1L (1000 nM) and either His-ROS1 or MBP-ROS1 (75 nM) were incubated during 15 min at 25°C with the labeled DNA duplex (10 nM). Protein-DNA complexes were detected as indicated above. (E) Interaction of APE1L with ROS1 by firefly luciferase complementation imaging assay in *Nicotiana benthamiana* leaves. Three independent experiments were done with similar results.
doi:10.1371/journal.pgen.1004905.g003

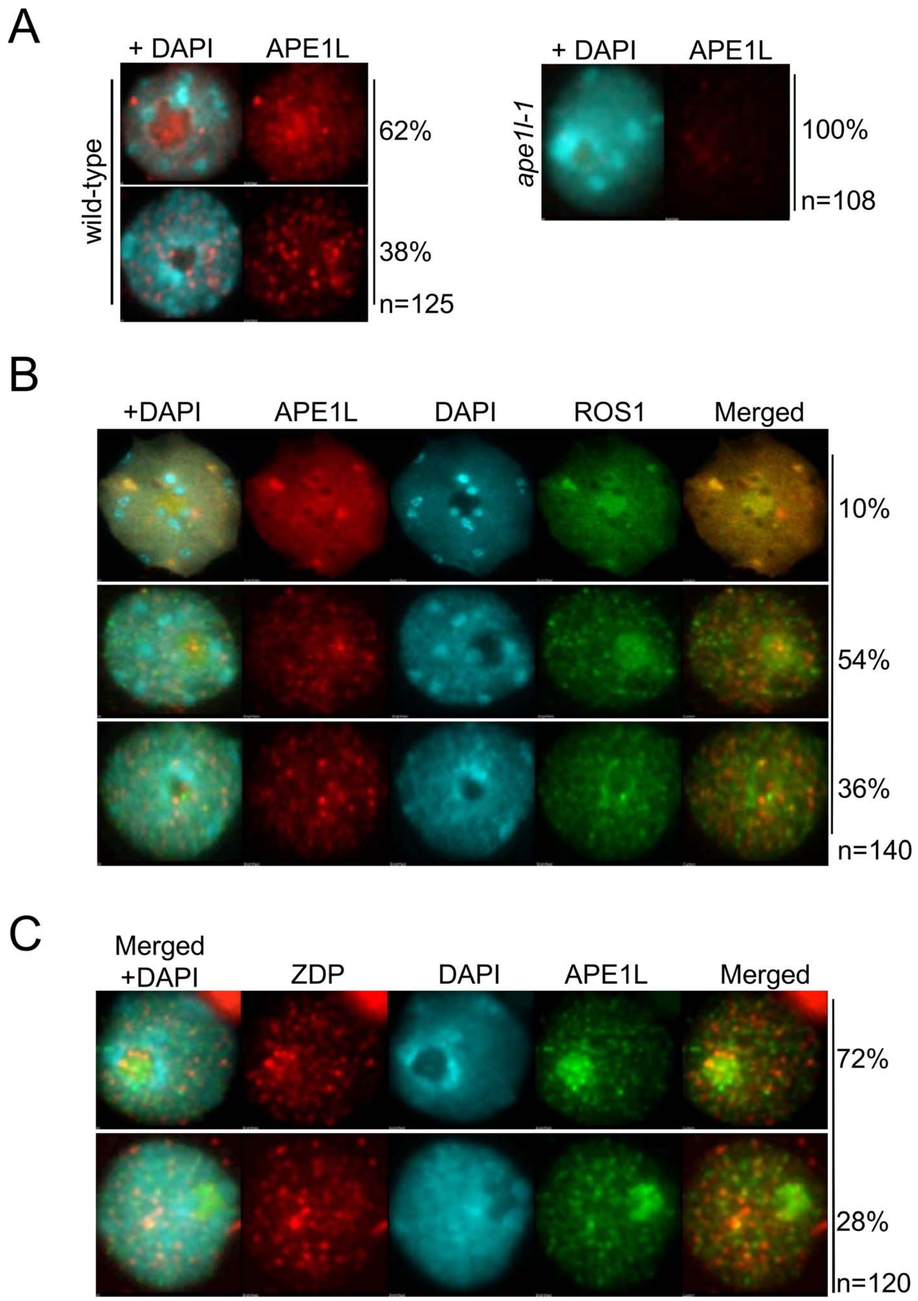

Fig. 4. Sub-nuclear localization of APE1L and co-localization with ROS1. (**A**) The nuclear distribution of APE1L was analyzed by immunostaining using anti-APE1L (red) in wild type and *ape1l-1* mutant plants. (**B**) Dual immunofluorescence using anti-APE1L (red) in transgenic lines expressing 3×Flag-ROS1 (green). (**C**) Dual immunofluorescence using anti-ZDP (red) in transgenic lines expressing APE1L-3×Flag (green). In all panels the DNA was stained with DAPI (blue). The frequency of nuclei displaying each interphase pattern is shown on the right.
doi:10.1371/journal.pgen.1004905.g004

co-localize in nucleoplasmic foci in approximately 28% of cells (Fig. 4C). Thus, APE1L co-localizes with components of the DNA demethylase machinery in distinct subnuclear structures in a subset of cells.

APE1L dysfunction causes genome-wide alterations in DNA methylation

To evaluate possible roles of APE1L in active DNA demethylation initiated by the ROS1 subfamily of DNA glycosylases/lyases, two T-DNA insertion lines were isolated for *APE1L* (S2A Fig.). RT-PCR analysis with *APE1L*-specific primers corresponding to the full-length open reading frame of the gene detected the expected product in wild-type plants in both the Ws and Col backgrounds, but not in *ape1l-1*, which is in Ws. In contrast, *ape1l-2* shows almost the same expression level as wild-type plants (S2B Fig.). Since the endonuclease ARP shows weak activity against the 3′-PUA blocking ends generated by ROS1 *in vitro*, we also isolated two T-DNA insertion lines for *ARP* (S2A Fig.) and confirmed by RT-PCR that they have a complete loss of mRNA expression (S2B Fig.). One of the mutants, *arp-1*, was used for further experiments.

To examine the general DNA methylation status in the *ape1l-1* and *arp-1* mutants, we compared the susceptibility of *5S* rDNA and 180-bp centromeric repeat regions to the restriction enzymes HpaII and MspI. These enzymes recognize the same site (CCGG), but HpaII cleavage is methylation-inhibited whereas methylation does not affect cleavage by MspI. DNA cleavage was assessed by Southern analysis. Similar to the *zdp-1* and *ros1-4* mutations, the *ape1l-1* or *arp-1* mutation does not affect the DNA methylation levels at the *5S* rDNA or 180-bp centromeric repeats (S3 Fig.), suggesting that the *ape1l-1* and *arp-1* mutants do not have changes in their overall DNA methylation patterns.

We performed whole genome bisulfite sequencing using DNA from 14-day-old *ape1l-1*, *arp-1*, *zdp-1* and their corresponding wild-type control plants. The CG methylation levels in wild type (Col-0) and *zdp-1* mutant are similar, but the CHG and CHH levels are mildly elevated in *zdp-1* (S4A Fig.). For *ape1l-1*, its overall genome methylation level in CG, CHG and CHH contexts is slightly higher than that in Ws (S4B Fig.). In total, we identified 6389 DMRs (differentially methylated regions) in *ape1l-1* mutant plants, including 3497 hyper-DMRs that have a significant increase in methylation and 2892 hypo-DMRs that have a significant reduction in methylation (S4C Fig.; S3 Table). In contrast, *arp-1* only affects methylation levels at 403 genomic regions, including 162 hyper-DMRs and 241 hypo-DMRs (S4C Fig.). 1559 hyper-DMRs and 612 hypo-DMRs were identified from *zdp-1* (S4C Fig.; S4 Table). The hyper-DMRs and hypo-DMRs identified in *ape1l-1*, *zdp-1* and *ros1-4* are evenly distributed along the five chromosomes (S4D Fig.). To determine whether *APE1L* and *ZDP* mutations affected DNA demethylation in specific genomic regions, we analyzed intergenic regions, transposable elements (TEs) outside of genes, TEs overlapping with genes and genic regions. Unlike *zdp-1*, *ros-1* mutants and *ros1-3;dml2-1;dml3-1* (*rdd*) triple mutants, which have less than 43% of hypermethylated (hyper-) DMRs distributed in gene regions, in *ape1l-1* and *arp-1* more than 60% of the hyper-DMRs are distributed in gene regions (S4E Fig.). In contrast, the percentages of hyper-DMRs distributed in TEs in *ape1l-1* and *arp-1* are lower than those in *zdp-1*, *ros-1* and *rdd* mutants (S4E Fig.). These data indicate that the APE1L and ARP mutations preferentially impact DNA demethylation of gene regions while the ZDP and ROS1 mutations have a greater impact on TE regions. The distribution patterns of classified hypo-DMRs are different from those of hyper-DMRs. The percentages of hypo-DMRs in gene regions are higher than 70% in *rdd*, *zdp-1* and *ape1l-1*. The *arp-1* has a low percentage of hypo-DMRs in gene regions but high percentage of hypo-DMRs in intergenic regions (S4E Fig.). The *ape1l-1* mutation affects CHG and CHH demethylation more profoundly than CG demethylation, both in gene regions or in TEs (S5A Fig.). We also examined the effect of *APE1L* mutation on TEs of different lengths and found that the *ape1l-1* mutation has a bigger impact on shorter genes but longer TEs (S5B and S5C Figs.). Unlike *ape1l-1*, *zdp-1* shows almost the same DNA methylation pattern for both gene regions and TEs (S5 Fig.).

Compared to the high level of overlap (70.9%) between *zdp-1* and *rdd* hyper-DMRs, less than 50% of the hyper-DMRs in *ape1l* overlap with those in *rdd* (S4C and S5D–S5E Figs.). One reason for this relatively low level of overlap may be the difference in genetic backgrounds; the *ape1l-1* mutant is in the Ws background whereas the other mutants are in Col. When the hyper-DMRs in *ros1-1* (C24 background) and *ros1-4* (Col-0 background) were compared, the overlap was also quite low (52%). For the hyper-DMRs, the level of overlap between *ape1l-1* and *zdp-1* is also very low (14%) (S5F Fig.) even though some loci do show hypermethylation in *ape1l-1* as well as *zdp-1* (S5G–S5J Fig.). These results are consistent with the notion that APE1L and ZDP largely represent two different mechanisms (AP endonuclease vs 3′-phosphatase) downstream of the DNA glycosylases/lyases, despite their redundant functions (as 3′-phosphatases).

ROS1 and *ZDP* mRNA levels are decreased in RdDM pathway mutants [17]. We examined the expression of *APE1L* and *ARP* in the RdDM mutants *nrpd1-3* and *nrpe1-11*, and found no substantial decreases in the mRNA levels in the mutants compared to Col (S6A Fig.). We also measured the expression levels of *ROS1* and *ZDP* in *ape1l-1* and *arp-1* mutants, and found that the expression levels are similar in the mutants compared to those in the Col or Ws wild type control plants (S6B Fig.). Also, unlike the *zdp-1* mutant, which is hypersensitive to MMS induced DNA damage, the *ape1l-1 and arp-1* mutants show a sensitivity level similar to that of wild-type plants (S7A Fig.).

The double mutant of *APE1L* and *ZDP* are embryonic lethal

To study the potential genetic interactions between *APE1L* and *ZDP*, we crossed *ape1l-1* and *zdp-1* mutant plants. Interestingly, we found that $ape1l^{+/-}zdp^{-/-}$ and $ape1l^{-/-}zdp^{+/-}$ plants produce many aborted seeds, suggesting that the double mutations of *APE1L* and *ZDP* are lethal (Fig. 5A). We grew the viable seeds, genotyped the seedlings, and found no $ape1l^{-/-}zdp^{-/-}$ plants (Table 1). The ratio of aborted seeds is 48.7% in self-pollinated $ape1l^{+/-}zdp^{-/-}$ plants and 26.5% in self-pollinated $ape1l^{-/-}zdp^{+/-}$ plants (S5 Table). Approximately seven days after pollination, the seeds fated to abortion show white color and plump phenotypes (S8A Fig.). The endosperm in those seeds fails to undergo cellularization and the growth of their embryos is arrested (S8B Fig.). Later, those seeds accumulate brown pigments and collapse.

The 48.7% seed abortion ratio of self-pollinated $ape1l^{+/-}zdp^{-/-}$ plants suggests that the lethality of this mutant may be maternally regulated. Also, because APE1L and ZDP may act downstream of ROS1 and DME and some of the characteristics of seed abortion in $ape1l^{+/-}zdp^{-/-}$ and $ape1l^{-/-}zdp^{+/-}$ mutants resemble those in the $dme^{+/-}$ mutant, we examined whether double mutations of *APE1L* and *ZDP*, like *dme* mutation, are also maternally lethal. If so, all seeds derived from a female gametophyte with *APE1L* and *ZDP* double mutations will abort irrespective of the paternal allele. We crossed $ape1l^{+/-}zdp^{-/-}$ (♀) with $ape1l^{+/+}zdp^{-/-}$ (♂) and the cross resulted in about 50% aborted seeds and 50% viable seeds. When we crossed them in a reverse direction, we observed 100% viable seeds (Fig. 5B and S5 Table). Furthermore, when we crossed $ape1l^{+/-}zdp^{-/-}$ (♀) plants with wild type plants, approximately 50% of the seeds aborted. These data indicate that $ape1^{+/-}$ $zdp^{-/-}$ mutant is indeed maternally lethal (Fig. 5B and S5 Table). However, the $ape1l^{-/-}zdp^{+/-}$ mutant is not maternally lethal, based on the fact that few seeds aborted when we crossed $ape1l^{-/-}zdp^{+/-}$ to Col or $ape1l^{-/-}zdp^{+/+}$ in either directions (Fig. 5B and S5 Table). This is consistent with its seed abortion ratio (26.5%) (S5 Table) and segregation ratio ($ape1l^{-/-}zdp^{+/+}$:$ape1l^{-/-}zdp^{+/-}$:$ape1l^{-/-}zdp^{-/-}$ = 0.98:2:0) (Table 1) when self pollinated.

We examined the morphology of aborting seeds from $ape1l^{+/-}$ $zdp^{-/-}$ and $ape1l^{-/-}zdp^{+/-}$ mutants using differential interference contrast microscopy. The major defects of aborting seeds are arrested embryo growth at the heart stage or earlier (S8B Fig.) and abnormal sizes of endosperm nuclei (S8C Fig.). In some aborting seeds, the embryos are invisible, indicating that the embryos are arrested very early in development. The aborting seeds of $ape1l^{+/-}$ $zdp^{-/-}$ and $dme^{+/-}$ both display arrested embryo growth. Unlike $ape1l^{+/-}zdp^{-/-}$ mutant seeds, $dme^{+/-}$ mutant seeds display clumps

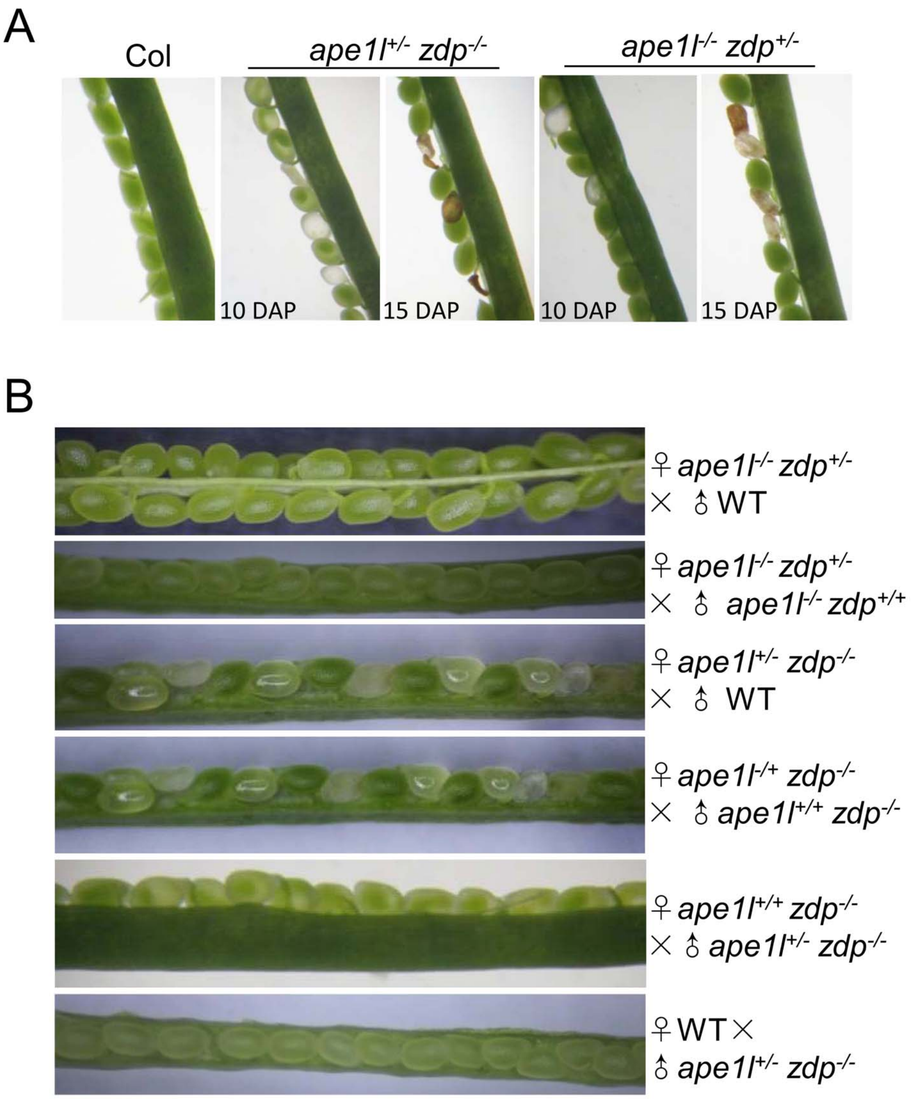

Fig. 5. Effects of *ape1l* and *zdp* double mutations on seed development. (**A**) Phenotypes of developing seeds in Col, *ape1l*$^{+/-}$*zdp*$^{-/-}$ and *ape1l*$^{-/-}$*zdp*$^{+/-}$ mutants. DAP: days after pollination. (**B**) Seed phenotypes from reciprocal crosses between different genotypes.
doi:10.1371/journal.pgen.1004905.g005

of unknown structures but there were no aberrant endosperm nuclei (S8B–C Fig.).

We noticed that the *ape1l*$^{+/-}$*zdp*$^{-/-}$ mutant has abnormal segregation ratio (4.07:1:0), which does not fit the expected segregation ratio of maternally lethal plant (1:1:0). Alexander staining and *in vitro* germination assay were carried out to examine the pollen development in different mutants. The *ape1l*$^{+/-}$ *zdp*$^{-/-}$ mutant showed defects in pollen development and germination (S9 Fig.), suggesting that the *ape1l*$^{+/-}$*zdp*$^{-/-}$ mutation not only leads to maternal lethality but also gives rise to paternal defects.

The double mutations of *APE1L* and *ZDP* cause DNA hypermethylation and down-regulation of imprinted genes in the endosperm

Maternal lethality phenotypes can be caused by aberrant expression of maternally imprinted genes and defects in the central cell or the endosperm [12,26,30]. *FWA* and *FIS2* are two well-studied maternally imprinted genes, and their maternal expression in the endosperm relies on active DNA demethylation initiated by DME [12,23]. We investigated whether the methylation of the *FWA* and *FIS2* promoters in endosperm tissues is affected by *APE1L* and *ZDP* double mutations (Fig. 6A). The *ape1l*$^{+/-}$*zdp*$^{-/-}$ plants were backcrossed to *zdp*$^{-/-}$ plants three times to minimize the Ws background. To examine the methylation levels of DME target genes in our mutants, we employed the method of Buzas et al. [36] where the DNA methylation specific restriction enzyme *McrBC* is used to digest DNA before doing q-PCR in seeds at 3 days post manual pollination. We found that after digestion with *McrBC*, the amount of DNA recovered from *FWA* and *FIS2* promoter regions (where is methylated in wild type leaf) was reduced in both *dme* and *ape1l*$^{-/-}$*zdp*$^{-/-}$ mutants compared with wild type, but there was no difference in the unmethylated *FWA* gene body region (Fig. 6A). These results indicate that the *ape1l*$^{-/-}$ *zdp*$^{-/-}$ endosperm has hypermethylation in *FWA* and *FIS2* promoter regions.

In order to measure the mRNA levels of *FWA* and *MEA* in Col and *ape1l*$^{-/-}$*zdp*$^{-/-}$ endosperms, we carried out real-time PCR and found that the expression levels of *FWA* and *MEA* but not the

Table 1. Genotypes of progeny from the self-cross of *ape1l*$^{+/-}$ *zdp*$^{-/-}$ and *ape1l*$^{-/-}$ *zdp*$^{+/-}$ plants.

ape1l$^{+/-}$ *zdp*$^{-/-}$ progeny	Total (213)
ape1l$^{+/+}$ *zdp*$^{-/-}$	171
ape1l$^{+/-}$ *zdp*$^{-/-}$	42
ape1l$^{-/-}$ *zdp*$^{-/-}$	0
Ratio	4.07:1:0
Expected ratio	1:1:0
Chi-square	76.92
Confidence	<0.01
ape1l$^{-/-}$ *zdp*$^{+/-}$ progeny	Total (180)
ape1l$^{-/-}$ *zdp*$^{+/+}$	59
ape1l$^{-/-}$ *zdp*$^{+/-}$	121
ape1l$^{-/-}$ *zdp*$^{-/-}$	0
Ratio	0.98:2:0
Expected ratio	1:2:0
Chi-square	6.25*10^-3
Confidence	>0.05

doi:10.1371/journal.pgen.1004905.t001

DME and *FIE* mRNAs are down-regulated in the *ape1l*$^{-/-}$*zdp*$^{-/-}$ mutant endosperm (Fig. 6B). To confirm and further analyze the *FWA* expression change, we introduced a *pFWA::ΔFWA-GFP* reporter into the *ape1l*$^{+/-}$*zdp*$^{-/-}$ and *ape1l*$^{-/-}$*zdp*$^{+/-}$ mutants by crossing the mutants with a transgenic line expressing the reporter [23]. Both *ape1l*$^{+/-}$*zdp*$^{-/-}$ and *ape1l*$^{-/-}$*zdp*$^{+/-}$ plants produce about 50% seeds defective in *pFWA::ΔFWA-GFP* expression (Fig. 6C–6D and S6 Table). To our surprise, *ape1l*$^{-/-}$*zdp*$^{+/-}$ mutant also produced 50% GFP-off seeds even though it is not maternally lethal and it produces about 75% viable seeds (Table 1). It turns out that hypermethylation of *pFWA::ΔFWA-GFP* promoter and silencing of FWA-GFP can occur in mutants which do not show maternal lethality. In addition, it seems that GFP-off seeds can be viable, so 75% viable seeds may be comprised of 50% GFP-on seeds and 25% GFP-off seeds. Taken together, our data suggest that DNA hypermethylation and down-regulation of imprinted genes occur and may be the cause of defects in the *ape1l*$^{-/-}$*zdp*$^{-/-}$ endosperm.

Discussion

Active DNA demethylation in plants is initiated by the ROS1 subfamily of 5-meC DNA glycosylases/lyases and presumably completed through a base excision repair pathway [2,37]. Previous work has reported that the 3′-phosphatase ZDP and the scaffold DNA repair protein XRCC1 also function in active DNA demethylation in *Arabidopsis* [17,38]. AP endonucleases are known to catalyze post-excision events during base excision repair. Our study here demonstrates that APE1L, one of the *Arabidopsis* AP endonucleases, functions in active DNA demethylation by processing β-elimination products of the bifunctional 5-meC DNA glycosylases/lyases and generating a 3′-OH group. APE1L-mediated reaction comprises a new branch of the DNA demethylation pathway downstream of ROS1, DME, DML2 and DML3 (Fig. 7). Our biochemical data show that APE1L has an additional, weak 3′-phosphatase activity, and thus may also function in the other branch, perhaps redundantly with ZDP, to process β, δ-elimination products. Interestingly, it has been recently reported that the wheat homolog of APE1L also possesses 3′-phosphatase and 3′-phosphodiesterase activities [39]. Our results suggest that APE1L not only functions downstream of ROS1, DML2 and DML3 in vegetative tissues to prevent DNA hypermethylation but also functions together with ZDP downstream of DME to control DNA demethylation and gene imprinting in the central cell and endosperm and is thus important for seed development.

Active DNA demethylation in mammals can be initiated through the deamination of 5meC by AID to generate thymine, or the oxidation of 5meC to generate 5-hydroxymethylcytosine (5hmC), and further to 5-formylcytosine (5fC) and 5-carboxycytosine (5caC) by the TET family of DNA dioxygenases [2,40–42]. 5fC and 5caC can be excised by the monofunctional DNA glycosylase TDG, whereas thymine can be removed by the monofunctional DNA glycosylase MBD4. Thus, a base excision repair pathway is required for completing the DNA cleavage and cytosine insertion steps during active DNA demethylation in mammals. Little is known about the DNA repair factors involved in active DNA demethylation in mammals, but it is likely that mammalian APE functions in active DNA demethylation downstream of the DNA glycosylases.

The *ape1l-1* mutation leads to DNA hypermethylation in thousands of genomic regions, indicating that APE1L is required for DNA demethylation in these regions in *Arabidopsis*. Like mutations in 5-methylcytosine DNA glycosylases/lyases such as ROS1, mutations in DNA repair enzymes downstream of these enzymes are expected to preclude active DNA demethylation and cause hypermethylation. Coordinating the DNA glycosylase/lyase and repair activity would be predicted to prevent an otherwise fatal accumulation of strand breaks throughout the genome [17]. APE1L and ROS1 physically interact *in vitro* and co-localize *in vivo*, strongly suggesting that these proteins form a complex which coordinates their activities. One may ask why the DNA demethylation pathway includes both lyase activity of ROS1 and AP endonuclease activity of APE1L. In a recent study, it was reported that Wheat APE1L has weak endonuclease activity but robust 3′-repair phosphodiesterase and 3′-phosphatase activities [43]. Even though we detected the endonuclease activity of *Arabidopsis* APE1L *in vitro*, it is possible that, like Wheat APE1L, *Arabidopsis* APE1L is weak in cleaving DNA backbone at AP sites when involved in DNA demethylation. In this case, the lysase activity of ROS1 is required for generating the DNA gap.

The *Arabidopsis* ARP endonuclease also processes the 3′-PUA generated by ROS1 *in vitro*, although this activity is much weaker than APE1L. Our whole genome bisulfite sequencing data identified only a small number of DMRs in the *arp-1* mutant. Therefore, ARP is unlikely to play a major role in DNA demethylation, at least under normal growth conditions.

Interestingly, we detected many genomic regions that are hypomethylated in the *ape1l-1* mutant. APE1L is a multifunctional enzyme; its APE and 3′-phosphatase activities may contribute to other DNA repair pathways in addition to active DNA demethylation, Thus, *APE1L* dysfunction may affect many DNA-related processes that directly or indirectly cause DNA hypomethylation. Compared to the *ros1-4* and *rdd* mutations, *ape1l* and *arp* mutations induce higher percentages of hypermethylation in genic regions, whereas the *zdp* mutation induces a higher percentage of hypermethylation in TEs. The mechanisms underlying this genomic specificity are unclear, but it is possible that APE1L and ARP function redundantly in the demethylation of TEs, such that mutating either one individually does not cause hypermethylation.

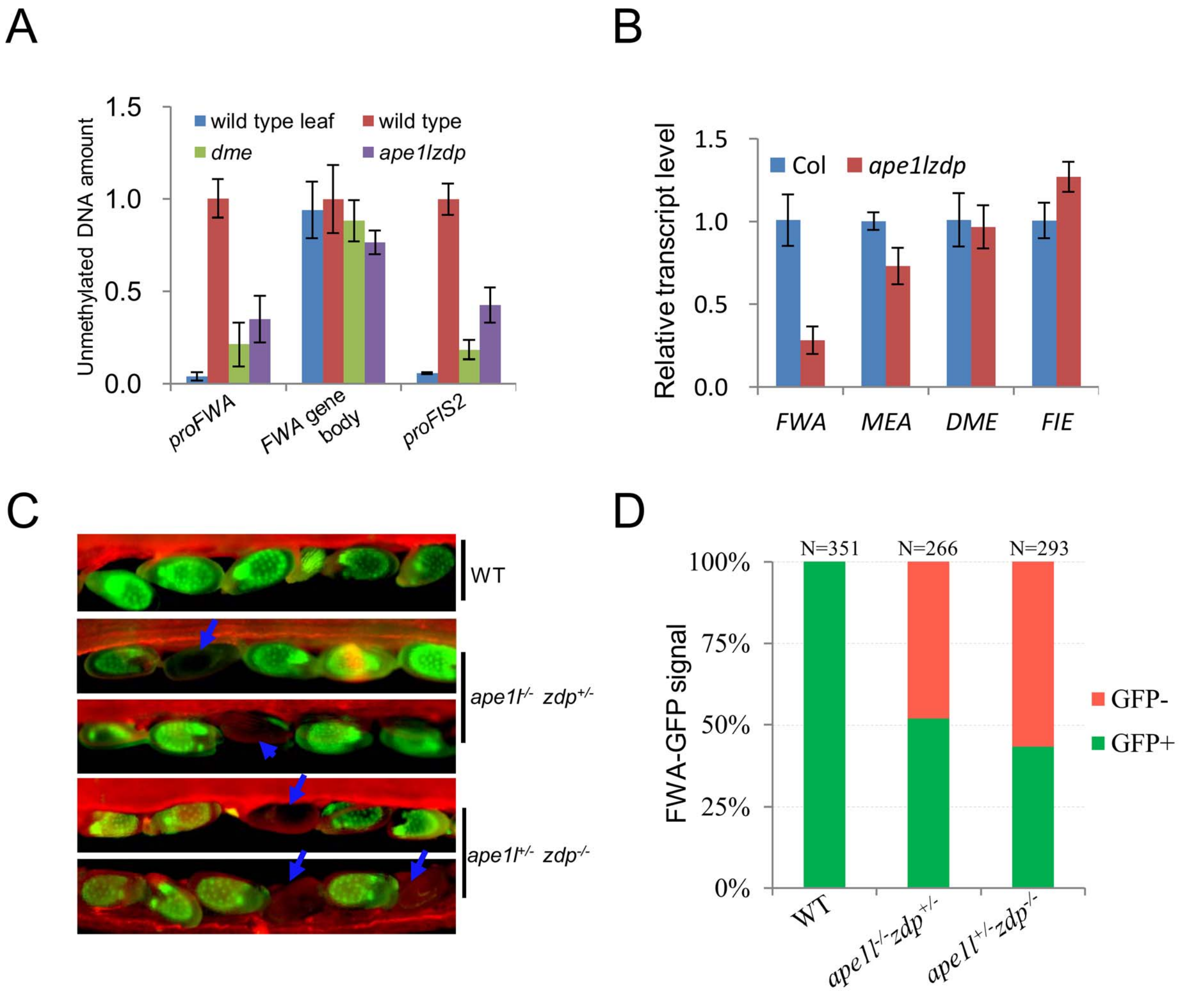

Fig. 6. Methylation levels and expression of imprinted genes. (**A**) Estimation of relative endosperm DNA methylation levels from wild type and *ape1lzdp* mutant in 3-days-after-pollination seeds. Equal amounts of seeds DNA were digested with *McrBC*, and undigested genomic DNA was used as a standard for absolute quantification of specific regions. The leaf sample was used as a control for the digest. Error bars represent standard error (n = 3) (**B**) qRT-PCR analysis of imprinted genes in wild type and mutant endosperm. The transcript levels were normalized against *ACT11* expression. Error bars represent standard error (n = 3). (**C**) Fluorescence images of *pFWA::ΔFWA-GFP* in Col, *ape1l*$^{+/-}$*zdp*$^{-/-}$ and *ape1l*$^{-/-}$*zdp*$^{+/-}$ plants. Arrows indicate seeds with silenced *FWA-GFP*. Images were captured at 4 days after pollination. (**D**) GFP phenotype of Col, *ape1l*$^{+/-}$*zdp*$^{-/-}$ and *ape1l*$^{-/-}$*zdp*$^{+/-}$ plants at 4 DAP. N indicates the number of ovules examined.
doi:10.1371/journal.pgen.1004905.g006

Unlike ZDP, which processes 3′-phosphate blocking ends and promotes the release of ROS1 from its products, APE1L converts both 3′-phosphate and 3′-PUA to 3′-OH, but does not increase the turnover of ROS1. Although both ZDP and APE1L interact with ROS1 *in vitro* and co-localize with ROS1 *in vivo*, ZDP and APE1L do not show extensive co-localization. It is possible that ZDP and APE1L exist mostly in two different protein complexes (Fig. 7). ZDP dysfunction caused DNA hypermethylation and transcriptional silencing of a luciferase reporter driven by the *RD29A* promoter, although the mutant phenotype was less severe than *ros1* mutants [17]. We hypothesized that at some DNA demethylation target regions, such as the *RD29A* promoter, the DNA glycosylases/lyases may use both β- and β, δ-elimination activities and thus require both APE and ZDP to process the intermediates and prevent transcriptional silencing. However, we found that the *ape1l-1* and *arp* mutations did not affect expression of the reporter gene (S7B Fig.). It is possible that APE1L may function redundantly with ARP and/or ZDP in demethylation of the *RD29A* promoter. *zdp* mutant showed sensitivity to MMS but *ape1l* and *arp* mutants are not sensitive to MMS probably because they carry out different reactions. In addition, APE1L, APE2 and ARP may play redundant roles in repairing MMS-induced DNA damage, such that the single mutation or double mutations are not sufficient to induce sensitivity to MMS.

The choice between the APE branch and the ZDP branch of the active DNA demethylation pathway depends on the elimination mechanism used by the DNA glycosylases/lyase enzymes. It is unclear when and where a DNA glycosylases/lyase employs β-elimination, β, δ-elimination, or both. Knowing which genomic regions depend on APE1L and which depend on ZDP for demethylation would be helpful. However, because the *zdp-1* and *ape1l-1* mutants are in different ecotypes, it is not ideal to compare the genomic regions targeted by the two different branches of the demethylation pathway.

The double mutations of *APE1L* and *APE2* are embryonic lethal, but not paternally or maternally lethal based on our results and the segregation ratio of selfed *ape1l*$^{+/-}$*ape2*$^{-/-}$ reported previously [31]. It is possible that the lethal phenotype caused by

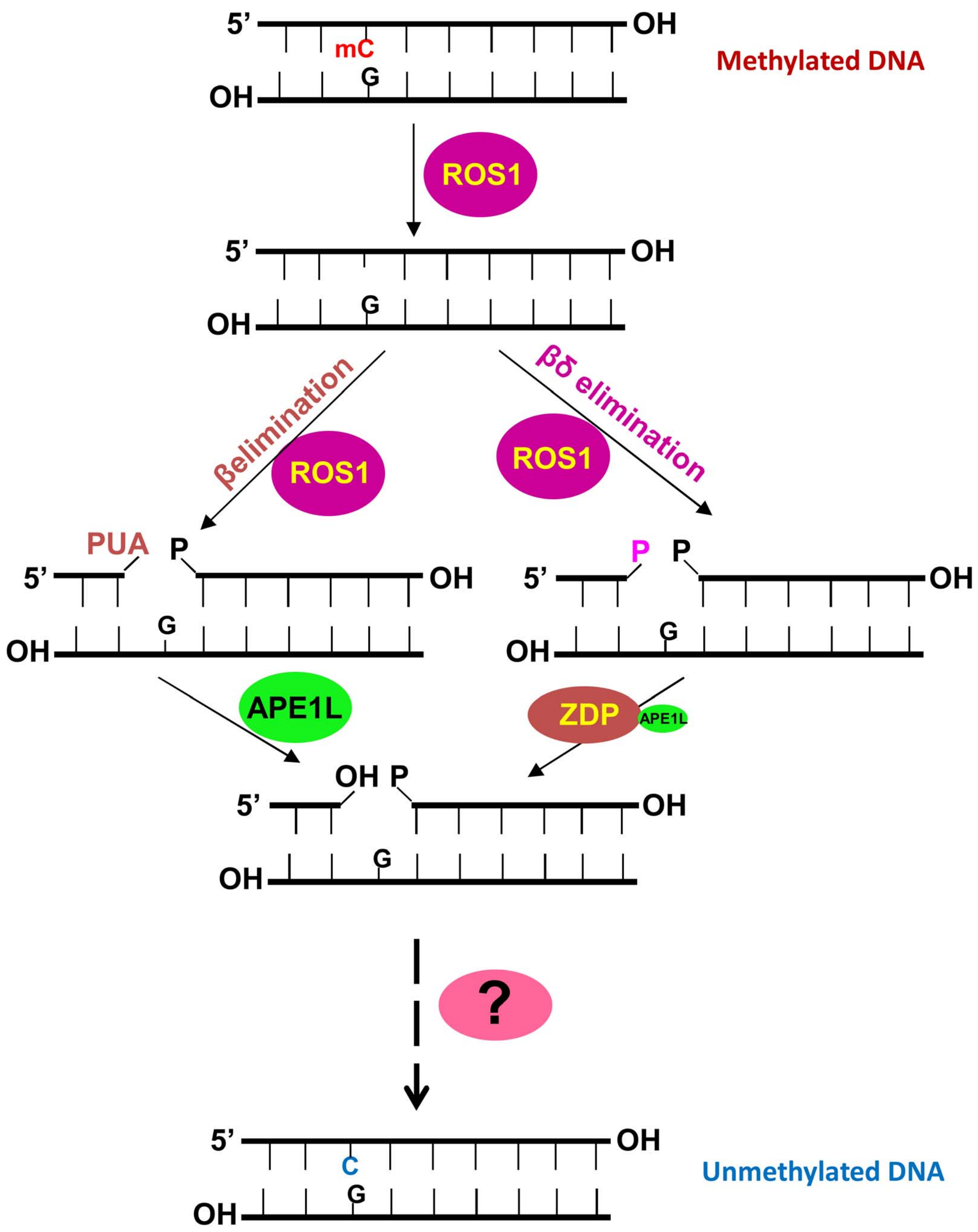

Fig. 7. A working model of the active DNA demethylation pathway in *Arabidopsis*. ROS1 (or DME, DML2 or DML3) is a bifunctional DNA glycosylase/lyase that removes the 5-methylcytosine base and then cleaves the DNA backbone at the abasic site using β- or β, δ -elimination, resulting in a gap with PUA or phosphate at the 3′ terminus which needs to be removed by APE1L or ZDP. Then the gap will be filled with an unmethylated cytosine nucleotide by an as yet unknown DNA polymerase and the processed strand will be sealed by a DNA ligase. The question mark represents unknown DNA polymerase and ligase enzymes.
doi:10.1371/journal.pgen.1004905.g007

APE1L and *APE2* double mutations reflect deficiencies of DNA repair. Interestingly, we found that the *ape1l*$^{+/-}$*zdp*$^{-/-}$ mutant shows a maternal lethality phenotype, which has been shown to occur in other mutants that are defective in DNA demethylation, such as the *dme* and *ssrp1* mutants [26,30]. Unexpectedly, only the *ape1l*$^{+/-}$*zdp*$^{-/-}$ mutant shows maternal lethality but the *ape1l*$^{-/-}$ *zdp*$^{+/-}$ mutant is not maternally lethal. As a result of maternal lethality, about 50% of seeds abort in *dme*$^{+/-}$ and *ape1l*$^{+/-}$*zdp*$^{-/-}$ mutants. In contrast, about 25% seeds abort in the *ape1l*$^{-/-}$*zdp*$^{+/-}$ mutant. All of the aborting seeds display embryos arrested at early growth stages presumably because an abnormal endosperm cannot support normal growth of the embryo. The morphology of aborted seeds in the *ape1l*$^{+/-}$*zdp*$^{-/-}$ and *ape1l*$^{-/-}$*zdp*$^{+/-}$ mutants is almost the same as that in the *ape1l*$^{+/-}$*ape2*$^{-/-}$ mutant, which is not maternally lethal and gives about 25% aborted seeds [31]. It is likely that the *ape1l*$^{+/-}$*ape2*$^{-/-}$ mutant is also defective

in DNA demethylation. Alternatively, this type of morphology (arrested embryo and aberrant endosperm) may reflect deficiencies of base excision repair.

It is likely that APE1L and ZDP function downstream of DME in the active DNA demethylation pathway that controls seed development. However, the aborting seeds in *ape1l*$^{+/-}$*zdp*$^{-/-}$ mutants have varied sizes of endosperm nuclei but the aborting seeds in *dme*$^{+/-}$ mutants have endosperm nuclei of uniform sizes. This phenotypic difference may arise because APE1L and ZDP have multiple functions in DNA demethylation and repair, whereas DME only participates in DNA demethylation. As in *dme*$^{+/-}$ mutants, the seed abortion phenotype in *ape1l*$^{+/-}$*zdp*$^{-/-}$ mutants is associated with the hypermethylation of the *FWA* promoter and the *MEA ISR*, and reduced *FWA* and *MEA* expression. Similar to the *ape1l*$^{+/-}$*zdp*$^{-/-}$ mutant, the *ape1l*$^{-/-}$*zdp*$^{+/-}$ mutant also produces about 50% GFP-off seeds, suggesting that these two types of mutants are similarly defective in DNA demethylation of imprinted genes. The phenotype of the *ape1l*$^{-/-}$*zdp*$^{+/-}$ mutant in pFWA-GFP silencing (50% GFP-off) and seeds viability (25% aborted and 75% viable) resembles that of the recently discovered *atdre2* mutant [44]. Some other factors beyond DNA demethylation or some dosage effects must be differentially involved in different types of mutants, leading to maternally lethality in some mutants but not in others, even though they are all defective in the expression of imprinted genes. In summary, our results show that APE1L and ZDP are important regulators of gene imprinting in plants, and suggest that DME-initiated active DNA demethylation in the central cell and endosperm employs both APE- and ZDP-dependent mechanisms.

Materials and Methods

Protein expression and purification

Full-length APE1L and APE2 cDNAs were subloned into pMAL-c2X (New England Biolabs) to generate MBP-APE1L and MBP-APE2 fusion proteins. The full-length ARP cDNA was subcloned into pET28a (Novagen) to generate a His-ARP fusion protein. Expression was induced in *Escherichia coli* BL21 (DE3) *dcm*$^{-}$ Codon Plus cells (Stratagene). MBP-APE1L and MBP-APE2 were purified by amylose affinity chromatography (New England Biolabs) and His-ARP was purified by affinity chromatography on a Ni^{2+}-nitrilotriacetic acid column (Amersham Biosciences). His-ROS1 and MBP-ROS1 were expressed and purified as previously described [15,34].

Site directed mutagenesis

Site directed mutagenesis of APE1L was performed using the Quick-Change II XL kit (Stratagene) according to the manufacturer's instructions. The N224D mutation was introduced into pMal-APE1L by using the oligonucleotides APE1LN212D_F4 and APE1LN212D_R4 (see S1 Table). The mutant sequence was confirmed by DNA sequencing, and the construct was used to transform *E. coli* strain BL21 (DE3) *dcm*$^{-}$ Codon Plus cells (Stratagene). Mutant protein was expressed and purified as described above for APE1L.

DNA substrates

Oligonucleotides used to prepare DNA substrates (see S2 Table) were synthesized by Integrated DNA Technologies [45] and purified by PAGE before use. Double-stranded DNA substrates were prepared by mixing a 5 μM solution of a 5′-fluorescein-labeled or 5′-Alexa Fluor-labeled oligonucleotide (upper strand) with a 10 μM solution of an unlabeled oligomer (lower strand). For preparation of 1-nt gapped DNA, a 5 μM solution of the corresponding 5′-labelled oligonucleotide was mixed with 10 μM solutions of unlabelled 5′-phosphorylated oligonucleotides P30_51 and CGR. Annealing reactions were performed at 95°C for 5 min, followed by slow cooling to room temperature.

Enzyme assays

To detect 5-meC DNA glycosylase/lyase activity, purified His-ROS1 (35 nM) was incubated at 30°C for 4 h with a Alexa Fluor-labeled DNA duplex (20 nM), containing a single 5-meC, in a reaction mixture containing 50 mM Tris–HCl pH 8.0, 1 mM DTT, 0.1 mg/ml BSA. In reactions containing APE1L, the mixture also included 200 mM NaCl and 1 mM $MgCl_2$. Reactions were stopped by adding 20 mM EDTA, 0.6% sodium dodecyl sulfate, and 0.5 mg/ml proteinase K, and the mixtures were incubated at 37°C for 30 min. DNA was extracted with phenol/chloroform/isoamyl alcohol (25:24:1) and ethanol precipitated at −20°C in the presence of 0.3 mM NaCl and 16 μg/ml glycogen. When the ROS1 reaction products were used as purified substrates for AP endonucleases (see below), samples were resuspended in 5 μl of distilled water. Otherwise, they were resuspended in 10 μl of 90% formamide, heated at 95°C for 5 min, and separated in a 12% denaturing polyacrylamide gel containing 7 M urea. Alexa Fluor-labeled DNA was visualized using the blue fluorescence mode of the FLA-5100 imager and analyzed using Multigauge software (Fujifilm).

The AP endonuclease activity was detected using a DNA substrate containing a synthetic AP site (tetrahydrofuran, THF) opposite G. The 3′-phosphatase activity was assayed on a 1-nt gapped substrate containing 3′-phosphate and 5′-phosphate ends. The 3′-phosphodiesterase activity was tested on purified ROS1 products, which contain a mixture of fragments with 3′-PUA and 3′-phosphate termini. In all assays, purified AP endonucleases were incubated with DNA substrates (20 or 40 nM) at 30°C for the indicated times in a reaction mixture containing 50 mM Tris–HCl pH 8.0, 200 mM NaCl, 1 mM DTT, 0.1 mg/ml BSA and 1 mM $MgCl_2$. Reactions were stopped and products analyzed as indicated above.

Pull down assays

Purified MBP alone or MBP-APE1L (200 pmol) in 100 μl of Column Buffer (20 mM Tris, pH 7.4, 1 mM EDTA, 1 mM DTT, 0.5% Triton X-100) was added to 100 μl of amylose resin (New England Biolabs) and incubated for 1 h at 4°C. The resin was washed twice with 600 μl of Binding Buffer (10 mM Tris, pH 8.0, 1 mM DTT, 0.01 mg/ml BSA). Purified His-ROS1 (15 pmol) was incubated at 25°C for 1 h with either MBP or MBP-APE1L bound to resin. The resin was washed twice with Binding Buffer. Bound proteins were analyzed by Western blot using antibodies against His_6 tag (Novagen).

Electrophoretic Mobility Shift Assay (EMSA)

EMSAs were performed using an Alexa Fluor-labeled duplex containing a gap flanked by 3′-phosphate and 5′-phosphate termini prepared as described above. The labeled duplex substrate (10 nM) was incubated with MBP-APE1L and/or His-ROS1 at the indicated concentrations in DNA-binding reaction mixtures (10 μl) containing 10 mM Tris HCl, pH 8.0, 1 mM DTT, 10 μg/ml BSA. After 15 min incubation at 25°C, reactions were immediately loaded onto 0.2% agarose gels in 1× Tris acetate/EDTA. Electrophoresis was carried out in 1× Tris acetate/EDTA for 40 min at 80 V at room temperature. Alexa Fluor-labeled DNA was visualized in a FLA-5100 imager and analyzed using MultiGauge software (Fujifilm).

Firefly luciferase complementation imaging assay

To investigate the interaction between APE1L and ROS1, two constructs was generated: APE1L-Cluc and ROS1-Nluc. The *BamHI* and *SalI* sites were used for cloning APE1L genomic DNA into pCAMBIA1300-CLUC vector. ROS1 was introduced to NLUC by In-Fusion HD Cloning Kit (Clontech). For protein interaction analysis, two combinatory constructs were transformed simultaneously into Nicotiana benthamiana leaves. To prevent the silencing of those genes, a virus p19 protein gene containing construct was transformed at the same time. After 3 d, 1 mM luciferin was sprayed onto the lower epidermis and kept in the dark for 5 min, then a CCD camera (1300B; Roper) was used to capture the fluorescence signal at 21°C.

Plant materials

Two T-DNA insertion mutants of the *APE1L* gene (*At3g48425*), INRA Flag240B06 and Salk_024194C, were used and they were referred to as *ape1l-1* and *ape1l-2* respectively. T-DNA insertions are present in the fifth exon and fourth intron of *ARP* in *arp-1* (SALK_021478) and *arp-2* (SAIL_866_H10) respectively. For all plants, seeds were sown on 1/2 MS plates containing 2% sucrose and 0.7% agar, stratified for 48 hours at 4°C and grown under long day conditions at 22°C. They were collected at 14 days or transplanted to soil.

Immunofluorescence

Immunofluorescence was performed in 2- to 3-week-old leaves as described by Pontes *et al.*, [46]. Nuclei preparations were incubated overnight at room temperature with rabbit anti-APE1L (anti-APE1L antibodies were generated by injecting rabbits with a recombinant full length APE1L protein that was purified by affinity chromatography), anti-ZDP [17] and mouse anti-Flag (F3165, Sigma). Primary antibodies were visualized using mouse Alexa 488-conjugated and rabbit Alexa-594 secondary antibody at 1:200 dilution (Molecular Probes) for 2 h at 37°C. DNA was counterstained using DAPI in Prolong Gold (Invitrogen). Nuclei were examined with a Nikon Eclipse E800i epifluorescence microscope equipped with a Photometrics Coolsnap ES.

RNA purification and real-time PCR

Total RNA was extracted from 2-week-old seedlings using the RNeasy Plant Mini Kit (QIAGEN). 2-µg RNA was used for the first-strand cDNA synthesis with the Super script III First-Strand Synthesis System (Invitrogen) for RT-PCR following the manufacturer's instructions. The cDNA synthesis reaction was then diluted five times, and 1 µl was used as template in a 20-µl PCR reaction with iQ SYBR Green Supermix (Bio-Rad). All reactions were carried out on the iQ5 Multicolour Real-Time PCR Detection System (Bio-Rad). The comparative threshold cycle (Ct) method was used for determining relative transcript levels (Bulletin 5279, Real-Time PCR Applications Guide, Bio-Rad), with *TUB8* as an internal control.

Whole genome bisulfite sequencing and data analysis

DNA was extracted from 2 g of 12-day-old seedlings grown in a growth chamber and sent to BGI (Shenzhen, China) for bisulfite treatment, library preparation, and sequencing.

Microscopy

Images of seed phenotypes were captured using an Olympus SZX7 microscope equipped with a Canon Powershot A640 camera. For cleared whole-mount observation, immature seeds, that are 8 days after pollination, were cleared using chloral hydrate, glycerol, and water (8 g: 1 ml: 2 ml) and photographed using a Leica DM6000 B differential interference contrast microscope equipped with a Leica DFC 425 camera. Fluorescence was detected with an Olympus BX53 fluorescence microscope equipped with an Olympus DP80 digital camera.

McrBC assay

The *McrBC* assay was performed according to Buzas et al [36]. Briefly, wild type and the *apel1*$^{+/-}$*zdp*$^{-/-}$ mutant were pollinated with Ler pollen. 3 days after pollination, pools of GFP-on and GFP-off seeds were selected under a dissecting fluorescence microscope and more than 300 seeds were used for DNA extraction. Genomic DNA concentration was measured by Nanodrop. Approximately 1 µg of DNA was digested with 1 µL of *McrBC* overnight at 37°C. After digestion, DNA methylation levels at the specific loci were determined by real-time PCR using absolute quantification against a 1:1 mixture of genomic DNA extracted from Col-0 and Ler leaves. Primers are listed in S1 Table.

RNA purification and real-time PCR using endosperm tissues

Female *ape1l*$^{+/-}$*zdp*$^{-/-}$ plants (Col-0) were crossed with male wild type plants. The endosperm plus seed coat fraction was collected for RNA purification using the Trizol method. DNAase treatment and LiCl precipitation were applied to remove DNA and polysaccharide contaminations, respectively. RNA was reverse transcribed into cDNA by the SuperScript III First-Strand Synthesis System (Invitrogen) with an oligo dT primer. Real-time PCR analysis was performed using SYBR Premix Ex Taq (TaKaRa) and CFX96 real-time system (Bio-Rad). *ACT11* was used as the internal control.

Accession numbers

We used whole-genome bisulfite sequencing to analyze the methylomes of *Ws*, *ape1l-1*, *arp-1 and zdp-1* mutant plants. The data set was deposited at NCBI (GSE52983).

Supporting Information

S1 Fig The DNA glycosylase/lyase activity of ROS1 is not increased in the presence of APE1L. Purified His-ROS1 (2 or 10 nM) was incubated in a reaction buffer lacking Mg^{2+} with a DNA substrate containing a 5-meC (20 nM) either in the absence or presence of purified MBP-APE1L (2, 10, 20 or 100 nM). Reactions were stopped after 16 hours and products were separated in a 12% denaturing polyacrylamide gel and detected by fluorescence scanning. (PDF)

S2 Fig Characterization of *APE* T-DNA mutants. (**A**) Mutant lines used and the positions of T-DNA insertions in each line. The green one is from the Ws background while black ones are from the Col background. blue boxes: exon; black boxes: intron: pink boxes: UTR. (**B**) RT-PCR results showing undetectable expression of *APE1L* and *ARP* transcripts in *ape1l-1*, *arp-1* and *arp-2* respectively. *ape1l-2* has a normal expression of *APE1L*. *TUB8* was used as a control.
(PDF)

S3 Fig Methylation status of *5S rDNA* and centromeric DNA repeats in the plants of different genotypes. Genomic DNA from plants of different genotypes was digested with the methylation sensitive enzyme *Hpa*II (CG and CHG methylation) or *Msp*I (CHG methylation), and hybridized with *5S rDNA*, or 180-bp centromeric repeat probes. *ddm1-10* was used as a hypomethylation control.
(PDF)

S4 Fig DNA methylome analysis by whole genome bisulfite sequencing in the different mutants. (**A–B**) Density of DNA methylation in *zdp-1* and *ape1l-1* mutants as compared to wild type plants. The densities of methylcytosines in each sequence context (CG, CHG, and CHH) across each chromosome in 50 kb segments are shown. (**C**) Numbers of hyper-DMRs and hypo-DMRs in the mutants examined. The percentages of hypermethylated regions that overlap with those in *rdd* mutant are shown on the right. (**D**) Distribution of hyper- and hypo-DMRs on the five chromosomes in the *ape1l-1*, *zdp-1* and *ros1-4* mutants. (**E**) Composition of the hypermethylated and hypomethylated genomic regions in the mutants examined.
(PDF)

S5 Fig DNA methylome analysis by whole genome bisulfite sequencing in wild-type and *ape1l-1* mutant plants. (**A**) Average methylation levels in gene and TE bodies. Genes or TEs were aligned from 2 kb upstream of transcription start sites to 2 kb downstream of transcription termination sites. (**B**) Average methylation levels in CG, CHG, or CHH context in genes of different lengths. (**C**) Average methylation levels in CG, CHG, or CHH context in TEs of different lengths. Gray-*ape1l-1*, green-Ws, blue-*zdp-1* and purple-Col. (**D–F**) Numbers of hypermethylated regions that are overlapping among or unique to the *ape1l-1*, *zdp-1*, *ros1-4 and rdd* mutants. (**G–J**) Examples of whole genome bisulfite sequencing data showing DNA hypermethylation in *rdd* and/or *zdp-1* and *ape1l-1* mutant plants. Red box-highlighted are regions that are hypermethylated in at least one of the mutants.
(PDF)

S6 Fig Expression analysis by qRT-PCR. (**A**) Expression of *ROS1*, *APE1L* and *ARP* in RdDM pathway mutants. (**B**) Expression of *ROS1* and *ZDP* in the *ape* and *nrpd1-3* mutants. The transcript levels were normalized against *ACT 11* expression. Error bars represent standard error (n = 3).
(PDF)

S7 Fig The sensitivity of *ros1-4*, *zdp* and *ape* mutants to MMS and the effect of *ape* mutant on the *RD29A-LUC* reporter gene. (**A**) Plants were grown for 14 days in MS nutrient agar plates containing 0 or 50 ppm MMS. (**B**) Effect of *ape1l-1* and *arp-1* on *RD29A-LUC* reporter gene. The reporter gene was introduced to *ape1l-1* and *arp-1* mutant plants by crossing. Seedlings grown in MS plate were imaged after cold treatment at 4°C for 24 hours.
(PDF)

S8 Fig Effects of *ape1l* and *zdp* double mutations on seed development. (**A**) A wild type seed, and an aborting seed showing endosperm over-proliferation (**B**) Cleared seed samples showing arrested embryo or invisible embryo in $ape1l^{+/-}zdp^{-/-}$, $ape1l^{-/-}zdp^{+/-}$ and $dme^{+/-}$ mutants as compared to seeds in Col. Images were captured at 8 DAP. (**C**) Images of cleared seed samples showing unequal sizes of endosperm nuclei in $ape1l^{+/-}zdp^{-/-}$ and $ape1l^{-/-}zdp^{+/-}$ mutants but not in Col and $dme^{+/-}$. Arrows indicate abnormally large endosperm nuclei. Images were captured at 8 DAP under a differential interference contrast microscope.
(PDF)

S9 Fig Effects of *ape1l* and *zdp* double mutations on pollen development. (**A–E**) Alexander staining of pollen grains from different genotypes. Viable pollen grains are stained in red. Nonviable pollen grains do not stain red and have different sizes and morphology compared with the viable grains. Bars = 100 μm. (**F**) *In vitro* germination of pollen from different genotypes. (*$P<0.05$).
(PDF)

S1 Table Primers and probes used in this study.
(DOCX)

S2 Table DNA sequence of oligonucleotides used as substrates.
(DOCX)

S3 Table Hyper- and Hypo-DMRs in *ape1l-1*.
(XLS)

S4 Table Hyper- and Hypo-DMRs in *zdp-1*.
(XLS)

S5 Table Seeds phenotype in self and reciprocal crosses between $ape1l^{-/-}zdp^{+/-}$, $ape1l^{+/-}zdp^{-/-}$ and Col.
(DOCX)

S6 Table The GFP phenotype in self crosses of $ape1l^{-/-}zdp^{+/-}$, $ape1l^{+/-}zdp^{-/-}$ and *pFWA-GFP*.
(DOCX)

Acknowledgments

We thank Terence M. Murphy for *aep1l-1* and *arp-1* seeds, Tetsuji Kakutani for *pFWA::ΔFWA-GFP* seeds, Rebecca A. Stevenson for technical assistance.

Author Contributions

Conceived and designed the experiments: WQ TRA JKZ. Performed the experiments: YL DCC WQ XZ. Analyzed the data: YL DCC WQ KT RRA. Wrote the paper: YL HZ TRA JKZ.

References

1. Law JA, Jacobsen SE (2010) Establishing, maintaining and modifying DNA methylation patterns in plants and animals. Nat Rev Genet 11: 204–220.
2. Zhu JK (2009) Active DNA demethylation mediated by DNA glycosylases. Annu Rev Genet 43: 143–166.
3. Zilberman D (2008) The evolving functions of DNA methylation. Curr Opin Plant Biol 11: 554–559.
4. Haag JR, Pikaard CS (2011) Multisubunit RNA polymerases IV and V: purveyors of non-coding RNA for plant gene silencing. Nat Rev Mol Cell Biol 12: 483–492.
5. Tariq M, Paszkowski J (2004) DNA and histone methylation in plants. Trends Genet 20: 244–251.
6. Borges F, Calarco JP, Martienssen RA (2012) Reprogramming the epigenome in Arabidopsis pollen. Cold Spring Harb Symp Quant Biol 77: 1–5.
7. Matzke M, Kanno T, Daxinger L, Huettel B, Matzke AJ (2009) RNA-mediated chromatin-based silencing in plants. Curr Opin Cell Biol 21: 367–376.
8. Zhang X, Yazaki J, Sundaresan A, Cokus S, Chan SW, et al. (2006) Genome-wide high-resolution mapping and functional analysis of DNA methylation in arabidopsis. Cell 126: 1189–1201.
9. Pikaard CS (2013) Methylating the DNA of the most repressed: special access required. Mol Cell 49: 1021–1022.
10. Zhang H, Zhu JK (2012) Active DNA demethylation in plants and animals. Cold Spring Harb Symp Quant Biol 77: 161–173.
11. Gehring M, Henikoff S (2008) DNA methylation and demethylation in Arabidopsis. Arabidopsis Book 6: e0102.
12. Gehring M, Huh JH, Hsieh TF, Penterman J, Choi Y, et al. (2006) DEMETER DNA glycosylase establishes MEDEA polycomb gene self-imprinting by allele-specific demethylation. Cell 124: 495–506.
13. Gong Z, Morales-Ruiz T, Ariza RR, Roldan-Arjona T, David L, et al. (2002) ROS1, a repressor of transcriptional gene silencing in Arabidopsis, encodes a DNA glycosylase/lyase. Cell 111: 803–814.
14. Ortega-Galisteo AP, Morales-Ruiz T, Ariza RR, Roldan-Arjona T (2008) Arabidopsis DEMETER-LIKE proteins DML2 and DML3 are required for appropriate distribution of DNA methylation marks. Plant Mol Biol 67: 671–681.
15. Morales-Ruiz T, Ortega-Galisteo AP, Ponferrada-Marin MI, Martinez-Macias MI, Ariza RR, et al. (2006) DEMETER and REPRESSOR OF SILENCING 1

encode 5-methylcytosine DNA glycosylases. Proc Natl Acad Sci U S A 103: 6853–6858.
16. Agius F, Kapoor A, Zhu JK (2006) Role of the Arabidopsis DNA glycosylase/lyase ROS1 in active DNA demethylation. Proc Natl Acad Sci U S A 103: 11796–11801.
17. Martinez-Macias MI, Qian W, Miki D, Pontes O, Liu Y, et al. (2012) A DNA 3′ phosphatase functions in active DNA demethylation in Arabidopsis. Mol Cell 45: 357–370.
18. Cordoba-Canero D, Morales-Ruiz T, Roldan-Arjona T, Ariza RR (2009) Single-nucleotide and long-patch base excision repair of DNA damage in plants. Plant J 60: 716–728.
19. Qian W, Miki D, Zhang H, Liu Y, Zhang X, et al. (2012) A histone acetyltransferase regulates active DNA demethylation in Arabidopsis. Science 336: 1445–1448.
20. Huh JH, Bauer MJ, Hsieh TF, Fischer RL (2008) Cellular programming of plant gene imprinting. Cell 132: 735–744.
21. Gehring M, Bubb KL, Henikoff S (2009) Extensive demethylation of repetitive elements during seed development underlies gene imprinting. Science 324: 1447–1451.
22. Hsieh TF, Ibarra CA, Silva P, Zemach A, Eshed-Williams L, et al. (2009) Genome-wide demethylation of Arabidopsis endosperm. Science 324: 1451–1454.
23. Kinoshita T, Miura A, Choi Y, Kinoshita Y, Cao X, et al. (2004) One-way control of FWA imprinting in Arabidopsis endosperm by DNA methylation. Science 303: 521–523.
24. Xiao W, Gehring M, Choi Y, Margossian L, Pu H, et al. (2003) Imprinting of the MEA Polycomb gene is controlled by antagonism between MET1 methyltransferase and DME glycosylase. Dev Cell 5: 891–901.
25. Ikeda Y (2012) Plant imprinted genes identified by genome-wide approaches and their regulatory mechanisms. Plant Cell Physiol 53: 809–816.
26. Choi Y, Gehring M, Johnson L, Hannon M, Harada JJ, et al. (2002) DEMETER, a DNA glycosylase domain protein, is required for endosperm gene imprinting and seed viability in arabidopsis. Cell 110: 33–42.
27. Schoft VK, Chumak N, Choi Y, Hannon M, Garcia-Aguilar M, et al. (2011) Function of the DEMETER DNA glycosylase in the Arabidopsis thaliana male gametophyte. Proc Natl Acad Sci U S A 108: 8042–8047.
28. Calarco JP, Borges F, Donoghue MT, Van Ex F, Jullien PE, et al. (2012) Reprogramming of DNA methylation in pollen guides epigenetic inheritance via small RNA. Cell 151: 194–205.
29. Ibarra CA, Feng X, Schoft VK, Hsieh TF, Uzawa R, et al. (2012) Active DNA demethylation in plant companion cells reinforces transposon methylation in gametes. Science 337: 1360–1364.
30. Ikeda Y, Kinoshita Y, Susaki D, Ikeda Y, Iwano M, et al. (2011) HMG domain containing SSRP1 is required for DNA demethylation and genomic imprinting in Arabidopsis. Dev Cell 21: 589–596.
31. Murphy TM, Belmonte M, Shu S, Britt AB, Hatteroth J (2009) Requirement for abasic endonuclease gene homologues in Arabidopsis seed development. PLoS One 4: e4297.
32. Zilberman MV, Karpawich PP (2007) Alternate site atrial pacing in the young: conventional echocardiography and tissue Doppler analysis of the effects on atrial function and ventricular filling. Pacing Clin Electrophysiol 30: 755–760.
33. Rothwell DG, Hickson ID (1996) Asparagine 212 is essential for abasic site recognition by the human DNA repair endonuclease HAP1. Nucleic Acids Res 24: 4217–4221.
34. Ponferrada-Marin MI, Roldan-Arjona T, Ariza RR (2009) ROS1 5-methylcytosine DNA glycosylase is a slow-turnover catalyst that initiates DNA demethylation in a distributive fashion. Nucleic Acids Res 37: 4264–4274.
35. Chen H, Zou Y, Shang Y, Lin H, Wang Y, Cai R, Tang X, Zhou JM (2008) Firefly luciferase complementation imaging assay for protein-protein interactions in plants. Plant Physiol. 146: 368–376.
36. Buzas DM, Nakamura M, Kinoshita T (2014) Epigenetic role for the conserved Fe-S cluster biogenesis protein AtDRE2 in Arabidopsis thaliana. Proc Natl Acad Sci U S A 111: 13565–13570.
37. Roldan-Arjona T, Ariza RR (2009) Repair and tolerance of oxidative DNA damage in plants. Mutat Res 681: 169–179.
38. Martinez-Macias MI, Cordoba-Canero D, Ariza RR, Roldan-Arjona T (2013) The DNA repair protein XRCC1 functions in the plant DNA demethylation pathway by stimulating cytosine methylation (5-meC) excision, gap tailoring, and DNA ligation. J Biol Chem 288: 5496–5505.
39. Joldybayeva B, Prorok P, Grin IR, Zharkov DO, Ishenko AA, et al. (2014) Cloning and characterization of a wheat homologue of apurinic/apyrimidinic endonuclease Ape1L. PLoS One 9: e92963.
40. Wu H, Zhang Y (2014) Reversing DNA methylation: mechanisms, genomics, and biological functions. Cell 156: 45–68.
41. Cortellino S, Xu J, Sannai M, Moore R, Caretti E, et al. (2011) Thymine DNA glycosylase is essential for active DNA demethylation by linked deamination-base excision repair. Cell 146: 67–79.
42. Rai K, Huggins IJ, James SR, Karpf AR, Jones DA, et al. (2008) DNA demethylation in zebrafish involves the coupling of a deaminase, a glycosylase, and gadd45. Cell 135: 1201–1212.
43. Joldybayeva B, Prorok P, Grin IR, Zharkov DO, Ishenko AA, et al. (2014) Cloning and characterization of a wheat homologue of apurinic/apyrimidinic endonuclease Ape1L. PLoS One 9: e92963.
44. Buzas DM, Nakamura M, Kinoshita T (2014) Epigenetic role for the conserved Fe-S cluster biogenesis protein AtDRE2 in Arabidopsis thaliana. Proc Natl Acad Sci U S A 111: 13565–13570.
45. Wohrmann HJ, Gagliardini V, Raissig MT, Wehrle W, Arand J, et al. (2012) Identification of a DNA methylation-independent imprinting control region at the Arabidopsis MEDEA locus. Genes Dev 26: 1837–1850.
46. Pontes O, Li CF, Costa Nunes P, Haag J, Ream T, et al. (2006) The Arabidopsis chromatin-modifying nuclear siRNA pathway involves a nucleolar RNA processing center. Cell 126: 79–92.
